# An individualized gene expression signature for prediction of lung adenocarcinoma metastases

**DOI:** 10.1002/1878-0261.12137

**Published:** 2017-10-10

**Authors:** Lishuang Qi, Tianhao Li, Gengen Shi, Jiasheng Wang, Xin Li, Sainan Zhang, Libin Chen, Yuan Qin, Yunyan Gu, Wenyuan Zhao, Zheng Guo

**Affiliations:** ^1^ College of Bioinformatics Science and Technology Harbin Medical University China; ^2^ Department of Bioinformatics Key Laboratory of Ministry of Education for Gastrointestinal Cancer School of Basic Medical Sciences Fujian Medical University Fuzhou China

**Keywords:** gene pair signature, genome lesions, lung adenocarcinomas, occult metastasis, prognosis

## Abstract

Our laboratory previously reported an individual‐level signature consisting of nine gene pairs, named 9‐GPS. This signature was developed by training on microarray expression data and validated using three independent integrated microarray data sets, with samples of stage I non‐small‐cell lung cancer after complete surgical resection. In this study, we first validated the cross‐platform robustness of 9‐GPS by demonstrating that 9‐GPS could significantly stratify the overall survival of 213 stage I lung adenocarcinoma (LUAD) patients detected with RNA‐sequencing platform in The Cancer Genome Atlas (TCGA; log‐rank *P *=* *0.0318, C‐index = 0.55). Applying 9‐GPS to all the 423 stage I‐IV LUAD samples in TCGA, the predicted high‐risk samples were significantly enriched with clinically diagnosed metastatic samples (Fisher's exact test, *P *=* *0.0015). We further modified the voting rule of 9‐GPS and found that the modified 9‐GPS had a better performance in predicting metastasis states (Fisher's exact test, *P *<* *0.0001). With the aid of the modified 9‐GPS for reclassifying the metastasis states of patients with LUAD, the reclassified metastatic samples presented clearer transcriptional and genomic characteristics compared to the reclassified nonmetastatic samples. Finally, regulator network analysis identified *TP53* and *IRF1* with frequent genomic aberrations in the reclassified metastatic samples, indicating their key roles in driving tumor metastasis. In conclusion, 9‐GPS is a robust signature for identifying early‐stage LUAD patients with potential occult metastasis. This occult metastasis prediction was associated with clear transcriptional and genomic characteristics as well as the clinical diagnoses.

**Abbreviations**
CI: confidence intervals
C‐index: concordance index
CNA: copy number aberrations
DE genes: differentially expressed genes
FDR: false discovery rate
GEO: Gene Expression Omnibus
GO: Gene Ontology
HR: hazard ratios
LUAD: lung adenocarcinomas
NSCLC: non‐small‐cell lung cancer
OS: overall survival
REO: relative expression ordering
RFS: recurrence‐free survival
TCGA: The Cancer Genome Atlas

## 1 Introduction

Among patients with non‐small‐cell lung cancer (NSCLC), which accounts for approximately 85% of all lung cancer cases, nearly 50% are lung adenocarcinomas (LUAD) (Chansky *et al*., 2009). For stage I LUAD patients with complete surgical resection, nearly 35–50% will relapse with poor prognoses (Siegel *et al*., 2015), which might be partially due to the high false‐negative rate of tiny distant metastases detection with current preoperative imaging techniques (Li *et al*., 2016; Pieterman *et al*., 2000). The problem of the high false‐negative rate also greatly limits the study on tumor metastasis mechanism because it will obscure the boundary between the primary tumors with metastasis and nonmetastasis and lead to very weak and irreproducible differential gene expression signals between the primary tumors with metastasis and nonmetastasis (Li *et al*., 2016). Therefore, many researches have been devoted to identifying metastasis prediction signatures based on gene expression profiles of primary NSCLC tissues (Choi *et al*., 2006; DiMeo *et al*., 2009; Xi *et al*., 2005). These signatures tend to show low accuracies in predicting nonmetastasis because clinically diagnosed nonmetastatic patients might harbor occult metastases (Li *et al*., 2016; Pieterman *et al*., 2000). Many other researches have been devoted to identifying prognostic gene signatures based on gene expression profiles of primary NSCLC tissues for auxiliary diagnosis of occult metastasis (Chen *et al*., 2011; Der *et al*., 2014; Lu *et al*., 2013; Ringner *et al*., 2016). However, most of the reported signatures are based on risk scores summarized from weighted expression levels of the signature genes, which are highly sensitive to measurement batch effects. It means that the analysis of a single sample requires the data of this sample to be normalized with a set of samples measured together, whereas the risk prediction of an individual sample will rely on the risk composition of other samples adopted for normalization together (Qi *et al*., 2016). Additionally, the gene expression measurements would also be greatly affected by sampling locations of the same tumor (Xu *et al*., 2015) and partial RNA degradation during sample preparation (Freidin *et al*., 2012), introducing further uncertainty for the risk score and risk classification of a patient.

Recently, we have reported a prognostic signature for stage I NSCLC based on the within‐sample relative expression orderings (REOs) of nine gene pairs (denoted as 9‐GPS) with the majority rule, which is highly robust in data measured by different microarray platforms (Qi *et al*., 2016). We have validated that 9‐GPS can be directly applied to individual samples measured by different laboratories with different microarray platforms, obviating the requirement of data normalization. Our previous studies have demonstrated that the within‐sample REOs are also rather robust against the differences in measurement principles of different platforms (Wang *et al*., 2015), tumor sampling locations (Xu *et al*., 2015), and partial RNA degradation during tumor sample preparation (Chen *et al*., 2017). In this study, using RNA‐sequencing data of LUAD samples derived from The Cancer Genome Atlas (TCGA), we firstly intended to validate the cross‐platform robustness of 9‐GPS previously trained and validated in microarray data. Then, we analyzed the association of high‐risk samples predicted by 9‐GPS with clinically diagnosed metastasis states and found that 9‐GPS based on the majority voting rule used in our original work (Qi *et al*., 2016) had suboptimum power in the identification of patients with metastases based on the gene expression of primary tissues.

Therefore, we reset a strict voting criterion for low‐risk identification requiring that at least seven gene pairs of 9‐GPS vote for low risk (denoted as 7/9‐GPS), and validated that 7/9‐GPS performed better in terms of sensitivity of metastasis detection, overall survival (OS), and 5‐year recurrence rate in both the TCGA RNA‐sequencing data and another two independent test data sets measured by two different microarray platforms. Then, we focused on providing evidences that 7/9‐GPS can aid in the identification of genomic and transcriptional characteristics of primary tumor tissues of patients with metastases by reclassifying the metastasis states of all patients with LUAD in TCGA. The high‐risk stage I patients predicted by 7/9‐GPS, compared with the low‐risk stage I patients, were also characterized by these genome lesions, greatly increasing the confidence of 7/9‐GPS for identifying occult metastasis of stage I patients in the clinical application (Liotta and Petricoin, 2012; Subramanian and Simon, 2010). Furthermore, the regulator network analysis identified *TP53* and *IGF1* with frequent genomic lesions in reclassified metastatic samples, which might play key roles in driving tumor metastasis.

## 2 Materials and methods

### 2.1 Data and preprocessing

The multiomics data of primary LUAD were downloaded from the TCGA data portal website (http://cancergenome.nih.gov/). For the 277 samples of stage I patients with recorded OS data, 64 samples with records of receiving adjuvant chemotherapy, radiotherapy, and/or target treatments were excluded from survival analysis. Of the remaining 213 samples of stage I patients (Table 1), 139 samples had records of recurrence data, which were used for recurrence risk analysis. Notably, of these 213 samples, only 21 samples were annotated with ‘None’ for any postoperative adjuvant treatments, while the other 192 samples were annotated with ‘Not available’ or ‘Unknown’, which were also used for survival analysis although a certain proportion of these patients might have received adjuvant therapies. This would be unlikely to result in false significant results because only if significantly more samples of the stage I patients who had received adjuvant therapies while simultaneously had occult metastases would be predicted to be at low risk, which would be unlikely to be the case. On the contrary, if some patients correctly predicted to be at high risk would actually have received adjuvant therapies with survival benefits, the significant prognostic difference between the high‐risk and low‐risk groups would be reduced or even lost, which may lead to false‐negative result for the signature validation. The clinical information of all the selected stage I samples is displayed in Table S1. Besides, all 423 stage I–IV primary samples of patients with LUAD (Table S2), including 266 samples of patients without metastases, 134 samples of patients with lymph node metastases, and 23 samples of patients with distal metastases, were used for metastatic and genomic analyses that did not need the survival data possibly confounded by various adjuvant therapies. As a high proportion of stage II–IV patients might be treated with adjuvant therapies, we did not perform survival analysis for these samples.

**Table 1:** The stage I LUAD samples used in this study

Data set	Stage I samples	Platforms
TCGA	213	Illumina HiSeq^a^
Test 1	301	Affymetrix Plus 2.0
GSE31210 (Okayama *et al*., 2012)	162	
GSE50081 (Der *et al*., 2014)	90	
GSE37745 (Botling *et al*., 2013)	29	
GSE31546	13	
GSE29013 (Xie *et al*., 2011)	7	
Test 2	28	Illumina HT‐12 V3.0
GSE29016 (Staaf *et al*., 2012)	28	

a HiSeq 2000 sequencing platform (Illumina).

For transcriptional data derived from HiSeq 2000 sequencing platform (Illumina, San Diego, CA, USA), the normalized count values processed by RSEM method were extracted and log 2‐transformed as the gene expression measurements. For gene mutation data of the 423 stage I–IV samples derived from the Illumina Genome Analyzer DNA Sequencing GAIIx platform, only the nonsynonymous mutations were included, and a discrete mutation profile including 17 821 genes was generated. Copy number aberrations (CNAs) of the 423 stage I–IV samples were downloaded from TCGA Firehose (http://gdac.broadinstitute.org/), which were processed with the GISTIC algorithm (Mermel *et al*., 2011) using the thresholds of 0.3 for copy number‐amplified regions and −0.3 for copy number‐deleted regions.

Six gene expression data sets of primary LUAD detected by microarray platforms, originally used for 9‐GPS validation in our previous study, were downloaded from the Gene Expression Omnibus (GEO, http://www.ncbi.nlm.nih.gov/geo/). Two tests were performed using the six data sets produced by two microarray platforms, including 301 stage I LUAD samples in test 1 (GSE31210, GSE50081, GSE37745, GSE31546, and GSE29013) generated by Affymetrix Plus 2.0 and 28 samples in test 2 (GSE29016) generated by Illumina HT‐12 V3.0. The two test data sets both had recorded OS data, while test 1 also had recorded recurrence data. The microarray data sets produced by Affymetrix U133A were not analyzed as only six gene pairs were measured in this platform. Additionally, GSE50081 data also include 33 primary tumor samples of LUAD patients with lymph node metastases and 94 primary tumor samples of LUAD patients without metastases. The raw mRNA expression data set was preprocessed using the Robust Multi‐array Average algorithm (Irizarry *et al*., 2003). Gene IDs were mapped to genes using the corresponding platform files. For each sample, the expression measurements of all probes corresponding to the same Gene ID were averaged to obtain a single measurement. Probes that did not match any Gene ID or matched multiple Gene IDs were deleted. All the samples used in this study were extracted from the primary tumors of LUAD patients with or without metastases.

The regulatory network data were integrated from the Pathway Commons (Cerami *et al*., 2011), SPIKE (Paz *et al*., 2011), SignaLink (Fazekas *et al*., 2013) databases, including 5800 regulators and 6695 targets (Babur *et al*., 2015). The functional pathways for enrichment analysis were downloaded from Gene Ontology (GO) (Ashburner *et al*., 2000) in November 2016.

### 2.2 Prognostic gene pair signature and survival analysis

**Figure 1:**
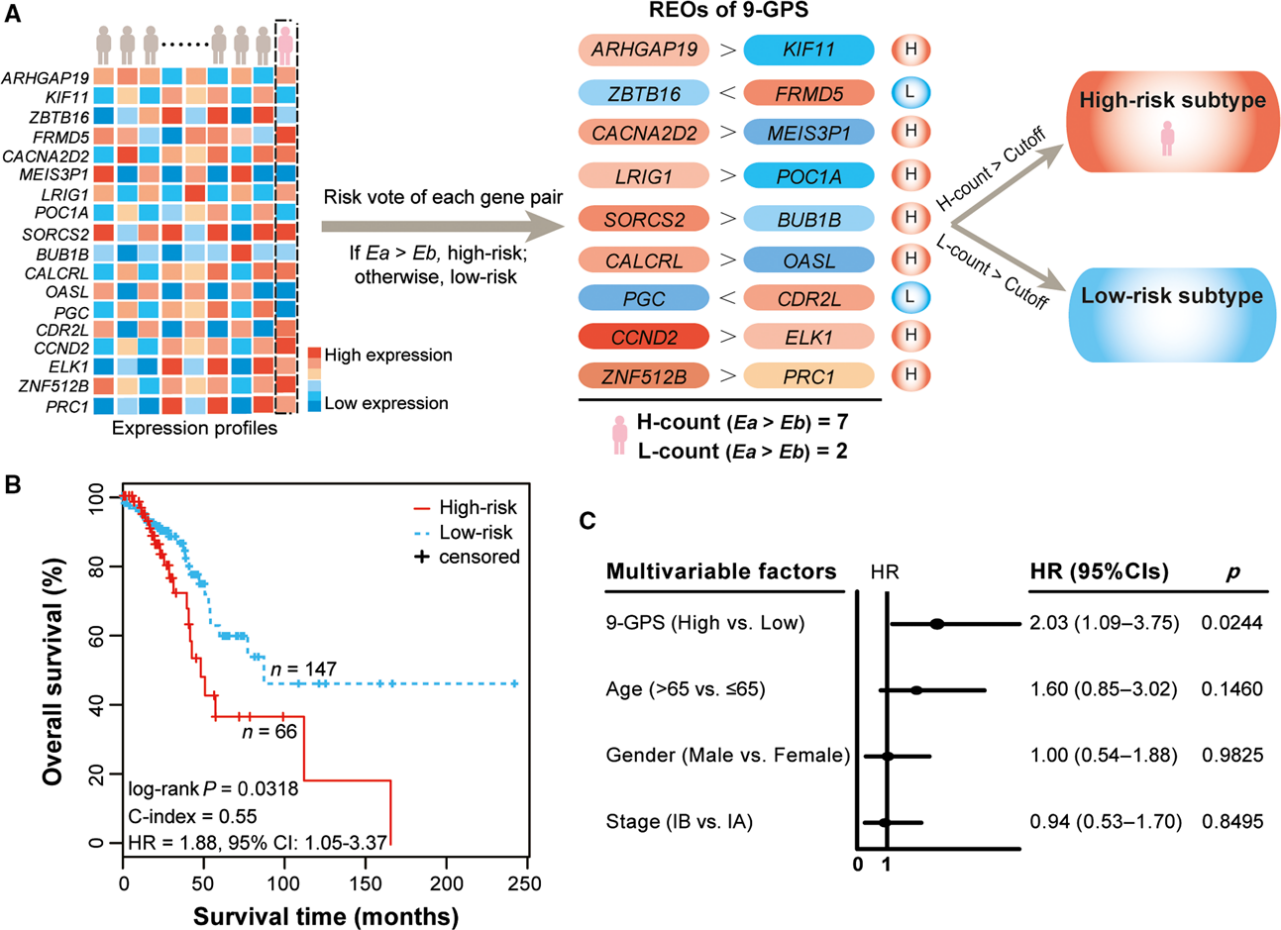
Prognostic 9‐GPS for risk classification and survival analyses for stage I lung adenocarcinoma samples in TCGA. (A) 9‐GPS for risk classification based on the within‐sample relative expression orderings (REOs) of the nine gene pairs with the majority voting rule. A sample is predicted to be at high risk if more than half of the nine gene pairs with the specific REOs (*Ea* > *Eb*); otherwise, it is predicted to be at low risk. The sample exemplar (pink) is predicted to be at high risk because the count of the specific REOs (*Ea* > *Eb*) in the individual is seven based on the majority voting rule. (B) The Kaplan–Meier curves of overall survival (OS) for stage I LUAD patients. Hazard ratio (HR) and 95% confidence interval (CI) were generated using univariate Cox regression models. (C) Multivariate Cox analyses for 9‐GPS with the majority voting rule, age, gender, and stage. Solid circles represent the HR for risk of death, and the open‐ended horizontal lines represent the 95% CI. The *P‐*value, HR, and CI were generated using multivariate Cox regression models.

The prognostic 9‐GPS signature consisting of nine gene pairs (Qi *et al*., 2016) is briefly described in Fig. 1A. Based on the majority voting rule, a cancer sample was determined to be at high (or low) risk if more than half of the REOs of the nine gene pairs in 9‐GPS voted for high (or low) risk. For each of the nine gene pairs, G*a* and G*b*, the REO pattern of *Ea *> *Eb (*or *Ea *< *Eb)* votes for high (or low) risk, where *Ea* and *Eb* represent the expression levels of G*a* and G*b*, respectively. In this study, we also evaluated the performance of 9‐GPS based on a strict voting criterion for low‐risk identification.

The OS was defined as the time from surgery to death or the final documented date (censored). The recurrence‐free survival (RFS) was defined as the time from surgery to recurrence or the final documented date (censored). Here, the 5‐year recurrence rate of patients was also used for survival analysis, which should be a better end point for prognosis study of early‐stage lung cancer due to high comorbidity during the usually long survival time. Survival curve was estimated using the Kaplan–Meier method and compared using the log‐rank test (Bland and Altman, 2004). We adopted the concordance index (C‐index) (Harrell *et al*., 1996) to estimate the predictive performance of a signature for patient survival. The multivariate Cox proportional hazards regression model was used to evaluate the independent prognostic value of the signature after adjusting for clinical factors including age, gender, and stage. Hazard ratios (HRs) and 95% confidence intervals (CIs) were generated using the Cox proportional hazards model.

### 2.3 Differential expression and functional enrichment analyses

Here, 11 642 genes with coefficient of variance > 0.10 were selected for differential expression analysis. Student's *t*‐test was conducted to extract significantly differentially expressed (DE) genes between two groups of samples. We used the GO function algorithm (Wang *et al*., 2012) to select GO pathways that significantly enriched with DE genes.

### 2.4 The genomic data analyses

Fisher's exact test was used to detect genes or genomic regions that had significantly different mutation or CNA frequencies between two subtypes. Here, we restricted the genomic analyses to the genes or genomic regions altered in more than 5% cancer samples. Spearman's rank correlation analysis was used to estimate the correlation of gene expression levels with gene mutations or CNAs.

The *P*‐values were adjusted using the Benjamini–Hochberg procedure for multiple testing to control the false discovery rate (FDR) (Benjamini and Hochberg, 1995). Significance was defined as *P *<* *0.05 or FDR < 0.05 for multiple testing. All statistical analyses were performed using the R 2.15.3 (http://www.r-project.org/).

## 3 Result

### 3.1 Prognostic performance of 9‐GPS in RNA‐sequencing data

9‐GPS for prognostic prediction of early‐stage NSCLC patients after complete surgical resection, as described in Fig. 1A, was previously trained and validated in multiple data sets measured by different laboratories with different microarray platforms (Qi *et al*., 2016). Here, we applied 9‐GPS to 213 stage I LUAD samples with RNA‐sequencing data in TCGA. Based on the within‐sample REOs of 9‐GPS, with the majority voting rule, 66 and 147 patients were classified into high‐ and low‐risk groups, respectively, with significantly different OS (log‐rank *P *=* *0.0318, HR* *=* *1.88, 95% CI: 1.05–3.37, C‐index* *=* *0.55, Fig. 1B). The multivariate Cox analysis showed that the 9‐GPS remained significantly associated with patient OS (*P *=* *0.0244, HR* *=* *2.03, 95% CI: 1.09–3.75, Fig. 1C) after adjusting for age (> 65 vs. ≤ 65), gender (male vs. female), and stage (IB vs. IA). The results validated that 9‐GPS extracted from microarray data could perform robustly in independent data assessed with the RNA‐sequencing platform, supporting the cross‐platform robustness of 9‐GPS for predicting OS of stage I LUAD.

### 3.2 Metastasis association of 9‐GPS in RNA‐sequencing data

**Figure 2:**
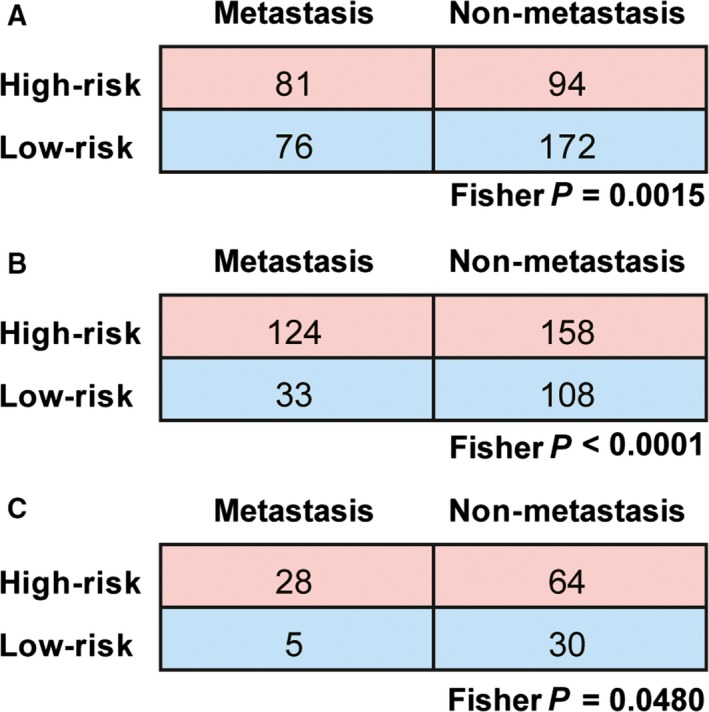
The association of high‐risk samples predicted by 9‐GPS with the primary tumor samples of patients with clinically diagnosed metastases. (A) The Confusion Matrix for the metastases prediction of 9‐GPS based on the majority rule in TCGA data. (B) The Confusion Matrix for the metastases prediction of 7/9‐GPS in TCGA data. (C) The Confusion Matrix for the metastases prediction of 7/9‐GPS in an independent data set (GSE50081). Fisher's exact test was used to compare the association.

To study the association of the risk classifications of samples predicted by 9‐GPS with clinically diagnosed metastasis states, 9‐GPS was applied to all the gene expression of 423 stage I–IV primary tumor samples in TCGA. Based on the majority voting rule, 175 and 248 patients were stratified into high‐risk and low‐risk groups, respectively. In the clinically diagnosed metastatic group, the proportion of samples identified as high risk was 51.59%, which was significantly higher than the corresponding proportion (35.34%) in the clinically diagnosed nonmetastatic group (Fisher's exact test, two‐sided *P *=* *0.0015, Fig. 2A). However, it is worth noting that quite a number of clinically diagnosed metastatic samples were classified as low‐risk samples, suggesting that the majority voting rule provided in our previous study (Qi *et al*., 2016) may have insufficient power to identify metastases.

### 3.3 Performance of 7/9‐GPS with a strict rule for low‐risk identification

Considering a signature as an auxiliary tool for clinical decisions, it should be reasonable to increase the sensitivity of metastasis identification while making conservative decisions on the identification of low‐risk patients who would be suggested to be treated with surgery only. Therefore, we tried to reset a strict criterion to identify low‐risk patients who are clinically diagnosed as nonmetastasis: A patient is determined to be at low risk only if all or significant more gene pairs vote for low risk; otherwise, high risk. When requiring that at least seven gene pairs of 9‐GPS vote for low‐risk determination, 56% of stage I LUAD samples were determined to be at high risk, which was closest to the clinically observed relapse rate of 35–50% for the stage I LUAD patients treated with curative surgery only (Siegel *et al*., 2015). Thus, we adopted this strict voting rule for low‐risk identification, denoted as 7/9‐GPS.

**Figure 3:**
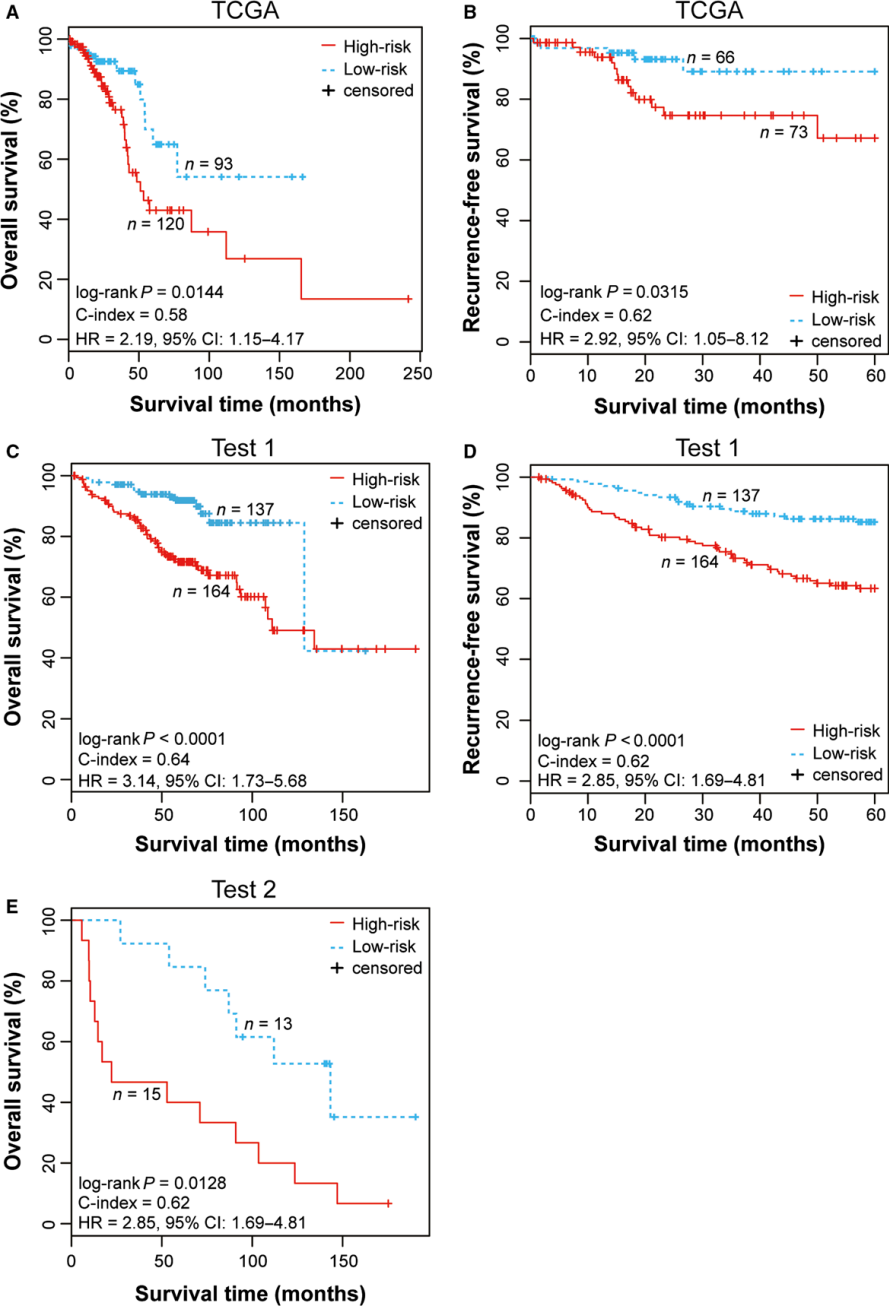
Prognostic performance of 7/9‐GPS with a strict rule for low‐risk identification. (A) The Kaplan–Meier curves of OS for 213 stage I LUAD samples in TCGA. (B) The Kaplan–Meier curves of recurrence‐free survival (RFS) for 139 stage I LUAD samples in TCGA. (C) The Kaplan–Meier curves of OS for 301 stage I LUAD samples in test 1. (D) The Kaplan–Meier curves of RFS for 301 stage I LUAD samples in test 1. (E) The Kaplan–Meier curves of OS for 28 stage I LUAD samples in test 2.

As expected, the sensitivity of metastasis detection based on the new voting rule of 7/9‐GPS increased greatly. Of the 157 primary tumor samples of patients with metastases in TCGA, 78.98% were identified as high‐risk samples, which was significantly higher than the corresponding frequency of 59.40% in the 266 primary tumor samples of patients without metastases (Fisher's exact test, two‐sided *P *< 0.0001, Fig. 2B). The result was validated in an independent data set (GSE50081): 84.85% of the 33 primary tumor samples of patients with metastases were identified as high‐risk samples by 7/9‐GPS, which was significantly higher than the corresponding frequency of 68.09% in the 94 primary tumor samples of patients without metastases (Fisher's exact test, one‐sided *P *=* *0.0480, Fig. 2C). Then, applying 7/9‐GPS to the 213 stage I samples in TCGA, we identified 120 high‐risk samples that had significantly shorter OS than the 93 samples identified as low‐risk samples (log‐rank *P *=* *0.0144, HR* *=* *2.19, 95% CI, 1.15–4.17, C‐index* *=* *0.58, Fig. 3A). Of these 213 stage I samples, 139 samples had records of recurrence information; thus, we also tested the prognostic performance of 7/9‐GPS for 5‐year recurrence rate of the patients. The result showed that the 5‐year recurrence rate for the 73 identified high‐risk samples was 0.33, which was significantly higher than the corresponding rate of 0.11 for the 66 samples identified as low‐risk samples (log‐rank *P *=* *0.0315, HR = 2.92, 95% CI = 1.05–8.12, C‐index = 0.62, Fig. 3B). When 9‐GPS based on the majority voting rule was applied, the 5‐year recurrence rate of the 36 samples predicted to be the high‐risk group was 0.37, which was higher, but not significantly, than the corresponding rate of 0.17 for the 103 low‐risk samples (log‐rank *P *=* *0.4962, HR = 1.40, 95% CI = 0.53–3.68, C‐index = 0.51, Fig. S1). Additionally, we found that the 5‐year recurrence rate of 54 high‐risk stage I patients identified by 7/9‐GPS but not by 9‐GPS was significantly higher than the corresponding rate of the 93 low‐risk stage I patients identified concordantly by 7/9‐GPS and 9‐GPS (log‐rank *P *=* *0.0252, Fig. S2A, while it was not significantly different from the 5‐year recurrence rate of the 66 high‐risk stage I patients identified concordantly by 7/9‐GPS and 9‐GPS (log‐rank *P *=* *0.5547, Fig. S2B). These results indicated that 7/9‐GPS performed better than 9‐GPS in identifying LUAD patients with occult metastases. The prognostic performance of 7/9‐GPS was also tested in the two test data sets used in our previous study (Qi *et al*., 2016), which were integrated from data detected by different laboratories with different microarray platforms. In the first test with 301 stage I LUAD samples integrated from five data sets generated by Affymetrix Plus 2.0, 7/9‐GPS identified 164 samples as high‐risk samples, which had significantly shorter OS than the 137 samples classified as low‐risk samples (log‐rank *P *<* *0.0001, HR = 3.14, 95% CI = 1.73–5.68, C‐index = 0.64, Fig. 3C). The 5‐year recurrence rate of the 164 high‐risk samples was 0.37, which was significantly higher than the corresponding rate of 0.15 for the 137 low‐risk samples (log‐rank *P *<* *0.0001, HR = 2.85, 95% CI = 1.69–4.81, C‐index = 0.62, Fig. 3D). In the second test with 28 stage I LUAD samples measured by Illumina HT‐12 V3.0, 15 high‐risk samples identified by 7/9‐GPS had significantly shorter OS than the 13 low‐risk samples (log‐rank *P *=* *0.0128, HR = 2.85, 95% CI = 1.69–4.81, C‐index = 0.62, Fig. 3E). 7/9‐GPS performed comparable with 9‐GPS in the first test (Fig. S3A,B), but better in the second test (Fig. S3C).

In general, 7/9‐GPS performed better than 9‐GPS in terms of sensitivity of metastasis detection, OS, and 5‐year recurrence rate.

### 3.4 Transcriptional characteristics of the reclassified metastatic samples revealed with the aid of 7/9‐GPS

**Figure 4:**
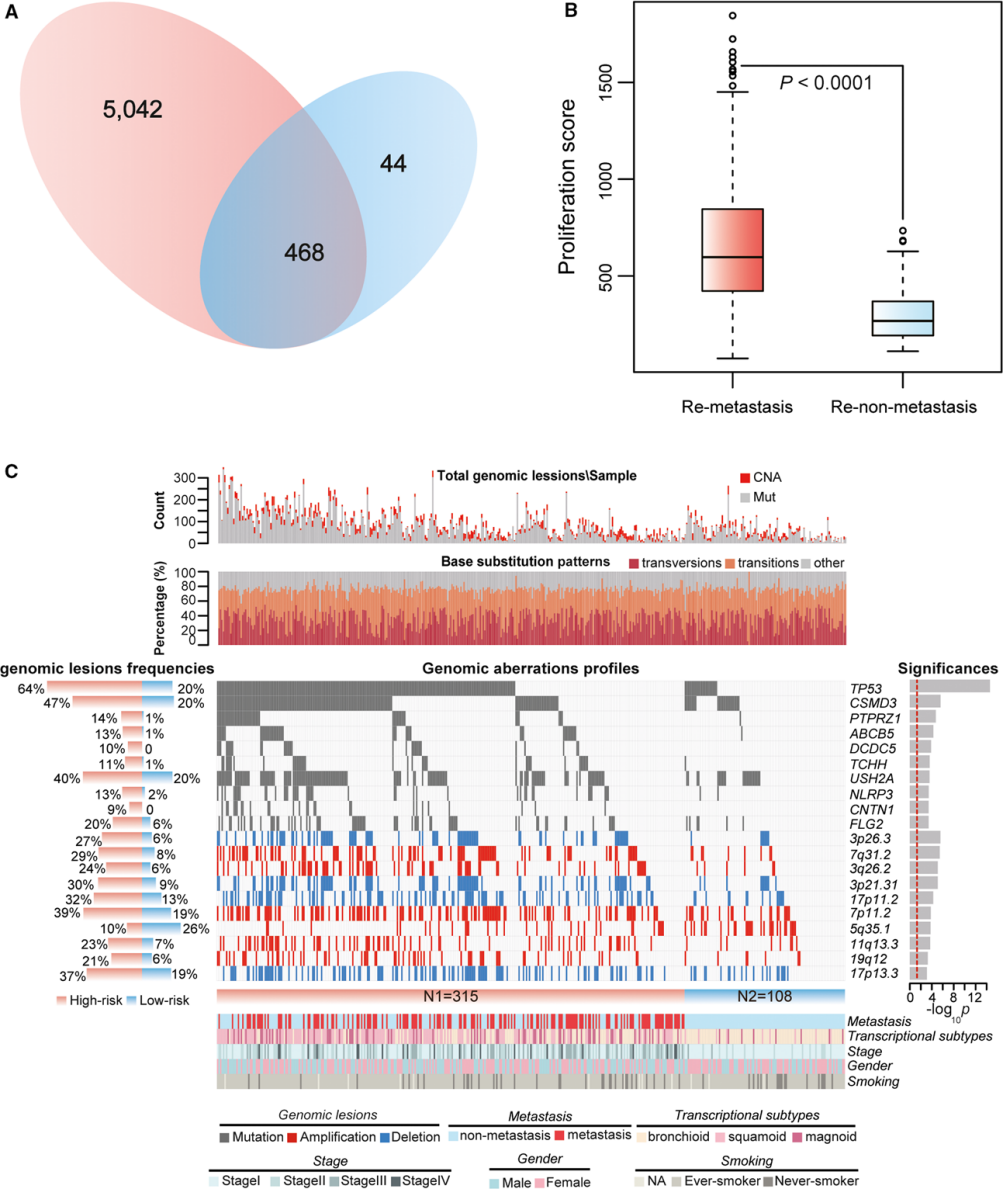
The transcriptional and genomic characteristics of the reclassified metastatic samples with the aid of 7/9‐GPS. (A) The overlap of the transcriptional differences between the primary tumor samples in the clinically diagnosed two metastatic groups and the reclassified two metastatic groups with the aid of 7/9‐GPS. The blue and pink circles represent the DE genes identified by the clinically diagnosed two metastatic groups and DE genes identified by the reclassified two metastatic groups, respectively. All the overlapped DE genes had the same dysregulated direction. (B) The boxplot of proliferation scores of the reclassified metastatic and nonmetastatic samples, respectively. (C) The genomic characteristics between the reclassified metastatic and nonmetastatic groups with the aid of 7/9‐GPS. Some genomic lesions between the two reclassified groups including ten gene mutations and ten chromosome regions with the most significant difference (Fisher's exact test, FDR < 0.05) are displayed. The frequencies of the two reclassified groups with lesions are shown at the left, and the significance of the frequency differences between the two groups is shown at the right. All the 48 genomic lesions that had different aberration frequencies between two reclassified groups are displayed in Table S4. The clinical information for clinically diagnosed metastasis states (yes/no), stage, gender, and smoking, and the total genomic lesions count, including mutation count and CNA count, mutation base substitution patterns, transcriptional subtypes for 423 stage I–IV samples, are also demonstrated.

Using Student's *t‐*test with 5% FDR control, we found only 512 DE genes between the primary tumor samples of patients with metastases and without metastases. With the aid of 7/9‐GPS, 108 primary tumor samples of patients without metastases, which were predicted as low‐risk samples by 7/9‐GPS, were kept as nonmetastatic samples, and the other 315 primary tumor samples that have happened metastases or were predicted as high‐risk samples by 7/9‐GPS were redefined as metastatic samples. Using Student's *t*‐test with 5% FDR control, we found that 5042 DE genes were detected between the redefined metastatic and nonmetastatic groups (Student's *t*‐test, FDR < 0.05). When compared the two DE gene lists (Fig. 4A), we found that 468 (91.41%) of the 512 DE genes between the clinically diagnosed two groups were also included in the DE genes identified after sample reclassification, and the dysregulation directions of the overlapped genes reached up to 100% (binomial test, *P *<* *0.0001). The clearer transcriptional differences between the two reclassified groups indicated that the reclassification of metastasis states of LUAD patients with the aid of 7/9‐GPS could capture more DE genes by reducing the influence of the samples with occult metastases. Functional enrichment analysis showed that the 5042 DE genes (denoted as metastasis‐related DE genes) identified after sample reclassification were significantly enriched in several pathways associated with tumor metastasis (hypergeometric distribution model, FDR < 0.05, Table S3), including ‘cell proliferation’ (Muller‐Tidow *et al*., 2001), ‘cell adhesion’ (Bremnes *et al*., 2002; Sin *et al*., 2011), ‘cell migration’ (Kim *et al*., 2009; Zheng *et al*., 2004), and ‘angiogenesis’ (Macchiarini *et al*., 1992).

Additionally, we used a proliferation signature consisting of 44 genes (Whitfield *et al*., 2006; Wu *et al*., 2013) to calculate a proliferation score for each sample that is the average expression of the 44 genes in that sample. The results showed that the reclassified metastatic samples identified by 7/9‐GPS had a significantly higher average proliferation score than the reclassified nonmetastatic samples (Student's *t*‐test, *P *<* *0.0001, Fig. 4B), which was concordant with the knowledge that high cell proliferation is closely related to tumor metastasis (Muller‐Tidow *et al*., 2001). Through unsupervised clustering analysis of primary LUAD gene expression profiles, reported by Wilkerson *et al*. (2012), the 423 stage I–IV LUAD samples were classified into three transcriptional subtypes: the bronchioid, the squamoid, and the magnoid subtypes. We found that the reclassified metastatic samples were significantly enriched in the squamoid and the magnoid subtypes, and the reclassified nonmetastatic samples were significantly enriched in the bronchioid subtype (chi‐square test, *P *<* *0.0001, Fig. 4C), which is characterized by low grade and the least invasion of tumor cells (Wilkerson *et al*., 2012). Similar result was observed in stage I samples in which the high‐risk samples identified by 7/9‐GPS were characterized by high proliferative capacities (Student's *t*‐test, *P *<* *0.0001, Fig. S4) and enriched in the squamoid and the magnoid subtypes (Fig. S5).

### 3.5 Genomics characteristics of the reclassified metastatic samples revealed with the aid of 9‐GPS

Using Fisher's exact test with 5% FDR control, we found no gene with significantly different mutation frequencies but only one chromosome region (7p11.2) with significantly different amplification frequencies between the 157 primary tumor samples of patients with metastases and 266 primary tumor samples of patients without metastases in TCGA (Fisher's exact test, FDR < 0.05). In contrast, we were able to find 21 genes with significantly different mutation frequencies and 27 genomic regions with significantly different CNA frequencies between the redefined metastatic and nonmetastatic groups (Fisher's exact test, FDR < 0.05). All the 48 genomic lesions are displayed in Table S4, while some are demonstrated in Fig. 4C. Impressively, 46 of the 48 genomic lesions had significantly higher frequencies of mutation or CNA in the reclassified metastatic group than in the reclassified nonmetastatic group (binomial distribution, *P *<* *0.0001). Additionally, we found that the median of the mutation count per sample for the reclassified metastatic samples was 80, which was significantly more than the corresponding median count (44) for the reclassified nonmetastatic samples (Student's *t*‐test, *P *<* *0.0001, Fig. 4C). Similarly, the median of the CNA count per sample for the reclassified metastatic samples was 18, significantly more than the corresponding median count (12) for the reclassified nonmetastatic samples (Student's *t*‐test, *P *<* *0.0001, Fig. 4C). Taken together, these results clearly showed that the reclassified metastatic samples suffered serious genomic instability. Many mutation genes are known to be related to tumor metastasis. For example, *TP53*, mutated in 63.81% of the 315 reclassified metastatic samples but only in 20.37% of the 108 reclassified nonmetastatic samples, could induce genomic instability (Negrini *et al*., 2010), aggravate tumor progression, and promote tumor metastasis (Marchetti *et al*., 1993; Reichel *et al*., 1994). For another example, a neural cell adhesion protein *CNTN1*, mutated in 8.57% of the reclassified metastatic samples but in none of the reclassified metastatic samples, could promote cancer cell invasion and metastasis (Shi *et al*., 2015; Yan *et al*., 2016). The expression levels of 608 genes within the 21 genomic regions with copy number gains or losses were positively correlated with their CNAs (Spearman's rank correlation, FDR < 0.05). Many genes in these chromosome lesions, such as *EGFR* (amp 7p11.2) (Eichler *et al*., 2010), *MET* (amp 7q31.2) (Breindel *et al*., 2013; Lutterbach *et al*., 2007), *KRAS* (amp 12p12.1) (Schmid *et al*., 2009), and *CACNA2D2* (del 3p21.31) (Warnier *et al*., 2015), are known to be related to tumor invasion and metastasis.

Notably, we found that 45 of the 48 genomic lesions characterizing the difference between the reclassified metastatic samples and nonmetastatic samples also had significantly different mutation or CNA frequencies between the stage I high‐risk and low‐risk samples identified by 7/9‐GPS (Fisher's exact test, FDR < 0.05, Fig. S5, Table S5), as shown in Fig. 4C. On the other hand, we found no genes or chromosome regions with significantly different mutation or CNA frequencies between the stage I high‐risk samples and primary tumor samples of patients with clinically diagnosed metastases. These results together supported that the stage I high‐risk samples identified by 7/9‐GPS might potentially carry occult metastases, which might obscure the differential genomic lesions between the clinically diagnosed metastatic and nonmetastatic samples.

Taken together, the reclassified metastatic samples are characterized by several genomic lesions related to LUAD metastasis.

### 3.6 Network analysis of ‘drivers’ for LUAD metastasis

Here, based on the regulatory relations among proteins documented in the integrated network, as briefly described in Materials and methods, we constructed a directed regulatory network by linking the 46 potential ‘drivers’ genomic lesions, which had significantly higher altered frequencies in the reclassified metastatic group, with the metastasis‐related DE genes between reclassified metastatic and nonmetastatic groups.

**Figure 5:**
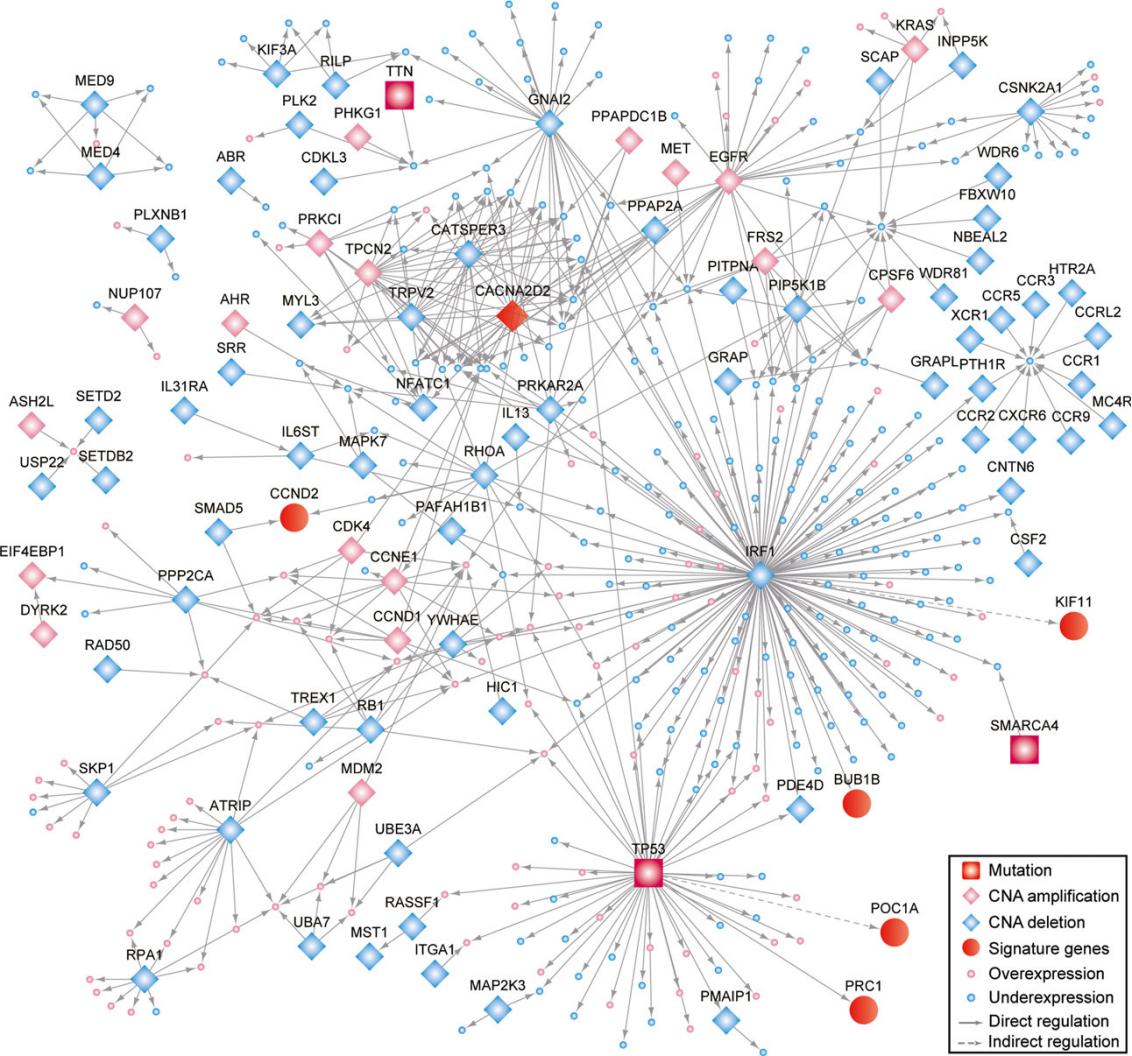
The regulatory network of ‘drivers’ for LUAD metastasis. The regulatory network includes ‘drivers’ for reclassified metastatic samples and metastasis‐related DE genes directly linked to the ‘drivers’. The nodes represent genes with genomic or transcriptional aberrations, and the edges represent the regulatory relations between ‘drivers’ and metastasis‐related DE genes. Three signature genes (*PRC1*,*CCND2*, and *BUB1B*) could be directly regulated by some genomic lesions, and another two signature genes (*KIF11* and *POC1A*) could be regulated by some genomic lesions indirectly. The remained signature genes were not annotated in the regulatory network. The functional pathways enriched with the metastasis‐related DE genes regulated by each of the ‘drivers’ in the regulatory network are displayed in Table S5.

The regulatory network included 85 ‘drivers’ (three mutated genes, 17 amplified genes, and 64 deleted genes) and 332 downstream metastasis‐related DE genes that were directly linked to the ‘driver’ genes. As shown in Fig. 5, two ‘driver’ genes, *TP53* and *IRF1*, appeared to regulate many metastasis‐related DE genes in the network. The 50 metastasis‐related DE genes regulated by *TP53* were significantly enriched in biological pathways related to metastasis (hypergeometric distribution model, FDR < 0.05, Table S6), including ‘apoptotic process’ (Moon *et al*., 2007), ‘cell growth’ (Muller‐Tidow *et al*., 2001), and ‘cell migration’ (Kim *et al*., 2009; Zheng *et al*., 2004). Another ‘driver’ gene, *IRF1*, was found to be deleted in 15.14% of the reclassified metastatic samples but only in 6.40% of the reclassified nonmetastatic samples. The 146 metastasis‐related DE genes regulated by *IRF1* were significantly enriched in several functional pathways (hypergeometric distribution model, FDR < 0.05, Table S6), such as ‘cell cycle’ (Muller‐Tidow *et al*., 2001), ‘activation of MAPK activity’ (Santarpia *et al*., 2012), and several pathways, including ‘cell–matrix adhesion’ (Sin *et al*., 2011) and ‘angiogenesis’ (Macchiarini *et al*., 1992), involved in tumor microenvironment, which were also related to cell migration and metastasis. The other metastasis‐related DE genes regulated by several ‘driver’ genes were also significantly enriched in several functional pathways related to tumor metastasis (hypergeometric distribution model, FDR < 0.05, Table S6). Notably, a signature gene *CACNA2D2* located in ‘3p21.31’ was deleted in 21.13% of the reclassified metastatic samples, but only in 5.56% of the reclassified nonmetastatic samples. The 18 metastasis‐related DE genes regulated by *CACNA2D2* were significantly enriched in several metastasis‐related pathways such as ‘MAPK signaling pathway’ (Santarpia *et al*., 2012). Three other signature genes (*PRC1*,* CCND2*, and *BUB1B*) could be directly regulated by some genomic lesions (Fig. 5), and another two signature genes (*KIF11* and *POC1A*) could be regulated by some genomic lesions indirectly (Fig. 5). The remained signature genes were not annotated in the regulatory network.

Taking together, the above regulatory network analyses indicated that *TP53* (Marchetti *et al*., 1993; Reichel *et al*., 1994), *IRF1* (Yuan *et al*., 2015). and some other genes such as *EGFR* (Eichler *et al*., 2010), *MET* (Breindel *et al*., 2013), *KRAS* (Schmid *et al*., 2009). and *CACNA2D2* (Warnier *et al*., 2015) with genomic lesions might play key roles in driving tumor metastasis. The results also suggested that the signature genes of 9‐GPS tended to be targets of molecular lesions in specific biological pathways for tumor cell proliferation, infiltration, and metastasis.

## 4 Discussion

In this study, we confirmed that the prognostic 9‐GPS extracted from microarray data for stage I LUAD patients could perform robustly for samples measured with the RNA‐sequencing platform. This result demonstrates the unique advantage of the cross‐platform robustness of the REO‐based signature. We proposed a hypothesis that the stage I LUAD patients with poor prognosis after complete surgical resection might harbor occult metastases, which was supported by the evidence that the predicted high‐risk samples were significantly enriched with the primary tumor samples of patients with metastases. However, we found that the majority voting rule provided in our previous study (Qi *et al*., 2016) might have insufficient power to predict metastasis states based on the gene expression of primary tumor samples. Considering the clinical actual needs, we proposed 7/9‐GPS based on a strict voting criterion for low‐risk identification and proved that 7/9‐GPS performed better than 9‐GPS originally based on the majority voting rule, in terms of sensitivity of metastasis detection, OS time, and 5‐year recurrence rate. With the aid of 7/9‐GPS, the primary tumor samples in the reclassified metastatic and nonmetastatic groups showed significantly different transcriptional and genomic characteristics related to tumor metastasis. Notably, most genomic lesions could not be detected between the primary tumor samples of LUAD patients with and without clinically diagnosed metastases, which should be due to some obscure clinical diagnosis for LUAD metastasis states. These results suggested that 7/9‐GPS could identify stage I LUAD patients who potentially have occult metastasis risk. However, the differences in some genomic lesions were still modest between the reclassified metastatic and nonmetastatic groups, which could be attributable to the heterogeneous of ‘driver’ genes in tumor samples, and different combinations of ‘driver’ genes might be more important (Ciriello *et al*., 2012). Finally, the regulatory network analysis revealed that genomic lesions of *TP53* and *IRF1* might play key roles in driving the metastasis of LUAD. Whether these findings can provide clues to new therapeutic targets merits further study.

Notably, about 20% of primary tumor samples of LUAD patients with clinically diagnosed metastases were identified as low‐risk samples by the signature. Although a certain percentage of metastatic samples could be false positives of clinical diagnosis (Pieterman *et al*., 2000), it also indicated that 7/9‐GPS might have insufficient power in metastasis identification. Therefore, 7/9‐GPS is intended to be an auxiliary tool for clinical metastasis diagnosis. Another limitation of this study is that some publicly available data sets were not used in this study because the patients in these data sets, such as GSE8894 (Lee *et al*., 2008) and GSE3141 (Bild *et al*., 2006), had not provided clear description on whether the patients had been treated with adjuvant treatment or not.

The qualitative nature of the within‐sample REOs makes the REO‐based signatures being highly robust against experimental batch effects and differences in probe designs used in different platforms (Guan *et al*., 2016). Consequently, the application of 9‐GPS to samples measured by different laboratories does not require data normalization, and thus, 9‐GPS can be applied at the individual level. It has been recognized that the subtle quantitative gene expression levels measured by current biotechnologies are quite error‐prone due to various factors such as the differences in reagents, reaction conditions, and operators (Leek *et al*., 2010), and data normalization methods, such as Combat (Johnson *et al*., 2007), DWD (Benito *et al*., 2004), and XPN (Shabalin *et al*., 2008), could distort real biological signals (Lazar *et al*., 2013). Therefore, qualitative REO‐based signatures would provide more reliable patient‐specific information for clinical application than quantitative signatures, as demonstrated in our previous study through comparing with the 15‐gene signature reported by Zhu *et al*. (2010). Here, we additionally evaluated two recently published quantitative prognostic signatures for NSCLC, including the malignancy risk gene signature reported by Chen *et al*. (2011) and the 16‐gene signature reported by Lu *et al*. (2013), both of which provided the risk scoring models and risk thresholds (see Supporting information). The malignancy risk gene signature classified all samples into high‐risk group when no *Z*‐score normalization was performed. The 16‐gene signature also could not predict prognosis of individual samples when no other samples were analyzed together for comparison. The requirement of between‐sample data normalization needs precollection of a set of samples for data normalization, and the risk prediction of an individual sample will rely on the risk composition of other samples adopted for normalization together (Qi *et al*., 2016; Xu *et al*., 2013). This provided further evidence that the type of quantitative signatures would be unfit to direct clinical settings, as reported in our previous study (Qi *et al*., 2016). Even when data normalization was performed, the two signatures also failed to predict OS of the 213 stage I samples with RNA‐sequencing data in TCGA (Fig. S6A,B), suggesting that this type of signatures could not perform robustly in data assessed with different platforms.

## 5 Conclusion

The REO‐based 7/9‐GPS is a true individual‐level prognostic signature, which is applicable for robustly identifying the stage I LUAD patients with potential occult metastases who should receive adjuvant drug treatments. It can also aid in the identification of genomic and transcriptional characteristics of patients with metastases.

## Author contributions

ZG conceived the idea. LQ conceived and designed the experiments and wrote the manuscript. TL designed the experiments. GS, JW, XL, SZ, LC, and YQ performed the experiments and analyzed the data. YG and WZ helped in interpreting the results and writing the manuscript. All authors approved the final version.

## Supporting information


**Fig. S1.** The Kaplan–Meier curves of recurrence‐free survival (RFS) for 139 stage I LUAD samples stratified by 9‐GPS based on the majority voting rule in TCGA.
**Fig. S2.** The survival analyses of the high‐risk samples identified by 7/9‐GPS but not by 9‐GPS and the risk samples concordantly by 7/9‐GPS and 9‐GPS in TCGA.
**Fig. S3.** Prognostic performance of 9‐GPS based on the majority voting rule in two test data sets.
**Fig. S4.** The boxplot of proliferation scores in the high‐risk and low‐risk samples identified by 7/9‐GPS, respectively.
**Fig. S5.** The genomic characteristics between the high‐ and low‐risk groups predicted by 7/9‐GPS in stage I LUAD patients.
**Fig. S6.** Prognostic performance of quantitative gene expression signatures in 213 stage I lung adenocarcinoma samples in TCGA.
**Table S1.** The clinical information of stage I LUAD samples in TCGA.
**Table S2.** The 423 stage I‐IV LUAD samples detected with multiple omic‐data in TCGA.
**Table S3.** The functional pathways enriched with metastasis‐related DE genes.
**Table S4.** The genomic characteristics between the reclassified metastatic and nonmetastatic groups with aid of 7/9‐GPS.
**Table S5.** The genomic characteristics between the stage I high‐risk and low‐risk samples identified by 7/9‐GPS.
**Table S6.** The functional pathways enriched with differentially expressed genes regulated by each ‘driver’ for reclassified metastatic samples.
