# Supplementary material for: An individualized gene expression signature for prediction of lung adenocarcinoma metastases
Source: Mol Oncol. 2017 Oct 10;11(11):1630–45. doi: 10.1002/1878-0261.12137 (PMC5663997; doi:10.1002/1878-0261.12137)
Supplement: Supplementary file 1 — Fig. S1. The Kaplan–Meier curves of recurrence‐free survival (RFS) for 139 stage I LUAD samples stratified by 9‐GPS based on the majority voting rule in TCGA. Fig. S2. The survival analyses of the high‐risk samples identified by 7/9‐GPS but not by 9‐GPS and the risk samples concordantly by 7/9‐GPS and 9‐GPS in TCGA. Fig. S3. Prognostic performance of 9‐GPS based on the majority voting rule in two test data sets. Fig. S4. The boxplot of proliferation scores in the high‐risk and low‐risk samples identified by 7/9‐GPS, respectively. Fig. S5. The genomic characteristics between the high‐ and low‐risk groups predicted by 7/9‐GPS in stage I LUAD patients. Fig. S6. Prognostic performance of quantitative gene expression signatures in 213 stage I lung adenocarcinoma samples in TCGA. Table S1. The clinical information of stage I LUAD samples in TCGA. Table S2. The 423 stage I‐IV LUAD samples detected with multiple omic‐data in TCGA. Table S3. The functional pathways enriched with metastasis‐related DE genes. Table S4. The genomic characteristics between the reclassified metastatic and nonmetastatic groups with aid of 7/9‐GPS. Table S5. The genomic characteristics between the stage I high‐risk and low‐risk samples identified by 7/9‐GPS. Table S6. The functional pathways enriched with differentially expressed genes regulated by each ‘driver’ for reclassified metastatic samples. [file MOL2-11-1630-s001.docx]

**An individualized gene expression signature for** **prediction of** **lung adenocarcinoma metastases**

Lishuang Qi^1†^, Tianhao Li^1†^, Gengen Shi^1^, Jiasheng Wang^1^, Xin Li^1^, Sainan Zhang^1^, Libin Chen^1^, Yuan Qin^1^,Yunyan Gu^1^, Wenyuan Zhao^1^, Zheng Guo^1,2*^

1 College of Bioinformatics Science and Technology, Harbin Medical University, Harbin 150086, China.

2 Department of Bioinformatics, Key Laboratory of Ministry of Education for Gastrointestinal Cancer,  School of Basic Medical Sciences, Fujian Medical University, Fuzhou, 350001, China

^†^These authors contributed equally to this work.

***Correspondence:** Zheng Guo, College of Bioinformatics Science and Technology, Harbin Medical University, Harbin 150086, China. E-mail: [guoz@ems.hrbmu.edu.cn](mailto:guoz@ems.hrbmu.edu.cn)

**Running title:** Metastasis-aided signature for lung adenocarcinoma

**Key words:** gene pair signature; prognosis; occult metastasis; genome lesions; lung adenocarcinomas

**The risk score calculation for the quantitative gene expression signatures**

The risk scores of samples based on the malignancy-risk gene signature were calculated according to the algorithm described in the original literature ([Chen *et al.*, 2011](#_ENREF_1)). Briefly, the expression data for each gene were first transformed to Z-scores by centering to the mean and scaling to the standard deviation, and then a risk score for a sample was calculated as a weighted sum of first principal component (a weighted average expression among the 94 malignancy-risk genes) derived from the transformed Z-scores of the 94 genes in the training data. Using the median of the risk scores of all samples in the training data as the risk threshold, which was calculated as being -0.3040, the samples were classified into the high- or low-risk group if their risk scores were higher (or equal to) or lower than the risk threshold.

The risk scores of samples based on the 16-gene signature were calculated according to the algorithm described in the original literature ([Lu *et al.*, 2013](#_ENREF_2)). Briefly, for each gene in the 16-gene signature, a score of 1 was given to a sample if the Cox coefficient from the training set was greater than 0 and the patient’s expression level was higher or equal to the median, or if the coefficient was less than 0 and the expression level was less than the median. The total risk score of each sample was computed for the 16 genes. Finally, samples were classified into two risk groups based on the median of the risk scores, which was calculated as being 8.

REFERENCES

Chen DT, Hsu YL, Fulp WJ, Coppola D, Haura EB, Yeatman TJ*, et al.* (2011). Prognostic and predictive value of a malignancy-risk gene signature in early-stage non-small cell lung cancer. *J Natl Cancer Inst* **103**(24)**:** 1859-1870.

Lu TP, Chuang EY, Chen JJ (2013). Identification of reproducible gene expression signatures in lung adenocarcinoma. *BMC Bioinformatics* **14:** 371.

**Table S1.** The clinical information of stage I LUAD samples in TCGA

| **Sample_id** | **OS (RFS)^** | **state** | **age (years)** | **gender** | **stage** | **Drug** |
| --- | --- | --- | --- | --- | --- | --- |
| TCGA-05-4249* | 50.77(50.77) | alive | 67 | Male | IB | NA |
| TCGA-05-4389* | 45.63(45.63) | alive | 70 | Male | IA | NA |
| TCGA-05-4403 | 19.27 | alive | 76 | Male | IB | UN |
| TCGA-05-4405* | 20.33(20.33) | alive | 74 | Female | IB | NA |
| TCGA-05-4417* | 15.17(15.17) | alive | 51 | Female | IB | NA |
| TCGA-05-4420* | 30.4(30.4) | alive | 41 | Male | IB | NA |
| TCGA-05-4422 | 12.17 | alive | 68 | Male | IB | UN |
| TCGA-05-4426* | 26.37(15.23) | alive(Rrcurrence) | 71 | Male | IB | UN |
| TCGA-05-4430* | 25.37(25.37) | alive | 59 | Female | IB | NA |
| TCGA-05-4433* | 24.33(24.33) | alive | 82 | Male | IB | NA |
| TCGA-05-5715 | 2.07 | alive | 69 | Female | IB | UN |
| TCGA-35-3615 | 0.47 | alive | 57 | Male | IB | UN |
| TCGA-35-4122 | 7.5 | alive | 69 | Male | IA | UN |
| TCGA-35-4123 | 6.07 | alive | 38 | Male | IA | UN |
| TCGA-38-4625 | 99.1 | alive | 66 | Female | IB | NA |
| TCGA-38-4631 | 11.8 | death | 72 | Female | IB | None |
| TCGA-38-A44F | 4.43 | alive | 80 | Male | IB | NA |
| TCGA-44-2655* | 44.13(44.13) | alive | 65 | Female | IA | None |
| TCGA-44-2656* | 47.63(47.63) | alive | 59 | Male | IB | None |
| TCGA-44-2657* | 45.03(45.03) | alive | 74 | Female | IB | NA |
| TCGA-44-2661* | 38.63(38.63) | alive | 69 | Female | IA | None |
| TCGA-44-2666* | 3.23(3.23) | death | 43 | Male | IB | None |
| TCGA-44-3398* | 38.77(38.77) | alive | 77 | Female | IA | NA |
| TCGA-44-3919* | 34.2(34.2) | death | 71 | Female | IA | NA |
| TCGA-44-5644* | 28.77(28.77) | alive | 51 | Female | IB | NA |
| TCGA-44-5645* | 28.4(28.4) | alive | 61 | Female | IA | NA |
| TCGA-44-6145* | 19.83(19.83) | alive | 62 | Female | IA | NA |
| TCGA-44-6147* | 28.17(28.17) | alive | 67 | Female | IA | NA |
| TCGA-44-6148* | 23.47(23.47) | alive | 60 | Male | IA | NA |
| TCGA-44-6775* | 23.5(23.5) | alive | 72 | Female | IB | NA |
| TCGA-44-6776* | 64.6(64.6) | alive | 60 | Female | IA | NA |
| TCGA-44-6778* | 62.13(62.13) | alive | 59 | Male | IA | NA |
| TCGA-44-7659* | 23.03(23.03) | alive | 70 | Male | IA | NA |
| TCGA-44-7661* | 12.2(11.17) | alive(Rrcurrence) | 69 | Female | IB | YES |
| TCGA-44-7662* | 7.27(7.27) | alive | 61 | Male | IB | NA |
| TCGA-44-7671* | 29.63(29.63) | alive | 64 | Male | IB | NA |
| TCGA-44-7672* | 13.93(13.93) | alive | 52 | Female | IA | NA |
| TCGA-44-8120* | 8.67(8.67) | alive | 58 | Male | IB | NA |
| TCGA-44-A479* | 16.2(16.2) | alive | 73 | Female | IB | NA |
| TCGA-44-A47A* | 15.53(13.33) | alive(Rrcurrence) | 78 | Female | IB | None |
| TCGA-44-A47B* | 9.57(9.57) | alive | 79 | Male | IB | NA |
| TCGA-44-A47G* | 11.7(11.7) | alive | 73 | Female | IA | NA |
| TCGA-44-A4SS* | 13.83(13.83) | alive | 73 | Male | IA | NA |
| TCGA-44-A4SU* | 13.63(8.67) | death(Rrcurrence) | 67 | Female | IA | None |
| TCGA-49-4486* | 77.27(68.17) | death(Rrcurrence) | 72 | Male | IA | None |
| TCGA-49-4487* | 28.5(23.23) | death(Rrcurrence) | 72 | Female | IA | None |
| TCGA-49-4488* | 28.97(21.13) | death(Rrcurrence) | 74 | Female | IA | None |
| TCGA-49-4501* | 47.37(18.17) | death(Rrcurrence) | 67 | Female | IB | YES |
| TCGA-49-4514* | 56.67(56.67) | alive | 79 | Female | IA | NA |
| TCGA-49-AAR0* | 158.83(158.83) | alive | 57 | Male | IA | NA |
| TCGA-49-AARE | 40.97 | death | 51 | Female | IA | UN |
| TCGA-49-AARN | 37.83 | death | 56 | Female | IA | UN |
| TCGA-49-AARO* | 125.3(117.37) | alive(Rrcurrence) | 39 | Female | IA | YES |
| TCGA-49-AARR* | 166.4(160.4) | alive(Rrcurrence) | 68 | Male | IA | None |
| TCGA-50-5931* | 14.47(14.17) | death(Rrcurrence) | 75 | Female | IB | None |
| TCGA-50-5939* | 15.33(15.33) | death | 85 | Male | IB | NA |
| TCGA-50-5944* | 25.47(25.47) | alive | 69 | Female | IA | NA |
| TCGA-50-5946* | 22.87(7.37) | alive(Rrcurrence) | 62 | Male | IA | YES |
| TCGA-50-6590 | 42.93 | death | 72 | Female | IB | UN |
| TCGA-50-6592 | 25.9 | death | 71 | Female | IB | UN |
| TCGA-50-6597* | 42.27(42.27) | death | 79 | Female | IB | NA |
| TCGA-50-7109* | 10.27(0.5) | death(Rrcurrence) | 60 | Male | IA | None |
| TCGA-50-8457* | 37.5(37.5) | alive | 63 | Female | IA | NA |
| TCGA-55-1592* | 23.37(15.07) | death(Rrcurrence) | 65 | Male | IA | None |
| TCGA-55-1595* | 49.3(49.3) | alive | 74 | Female | IA | NA |
| TCGA-55-5899 | 31 | alive | 58 | Male | IA | UN |
| TCGA-55-6543 | 14.5 | alive | 60 | Female | IA | UN |
| TCGA-55-6642 | 81.63 | alive | 63 | Male | IB | UN |
| TCGA-55-6969 | 41.3 | alive | 52 | Male | IB | UN |
| TCGA-55-6971 | 46.67 | alive | 59 | Female | IB | UN |
| TCGA-55-6972 | 54.4 | death | 72 | Male | IB | UN |
| TCGA-55-6980 | 70.3 | alive | 56 | Male | IA | UN |
| TCGA-55-6985 | 41.1 | alive | 58 | Female | IB | UN |
| TCGA-55-6986* | 108.7(108.7) | alive | 74 | Female | IB | NA |
| TCGA-55-6987* | 71.23(71.23) | alive | 77 | Male | IA | NA |
| TCGA-55-7281 | 29.07 | alive | 70 | Female | IA | UN |
| TCGA-55-7570 | 27.47 | alive | 60 | Male | IA | UN |
| TCGA-55-7573 | 16.23 | alive | 72 | Female | IA | UN |
| TCGA-55-7576 | 22.33 | alive | 54 | Male | IB | UN |
| TCGA-55-7724* | 23.5(23.5) | alive | 76 | Female | IB | NA |
| TCGA-55-7725 | 14.73 | alive | 68 | Female | IA | UN |
| TCGA-55-7726 | 21.73 | alive | 72 | Female | IA | UN |
| TCGA-55-7728 | 23.47 | alive | 64 | Female | IB | UN |
| TCGA-55-7815 | 25.77 | alive | 76 | Male | IB | UN |
| TCGA-55-7903* | 18.9(18.9) | alive | 64 | Male | IA | NA |
| TCGA-55-7995 | 29.63 | alive | 73 | Female | IA | UN |
| TCGA-55-8085* | 30.13(30.13) | alive | 64 | Male | IA | NA |
| TCGA-55-8087* | 15.4(15.4) | alive | 59 | Female | IB | NA |
| TCGA-55-8089 | 23.4 | death | 56 | Male | IA | UN |
| TCGA-55-8090* | 19.93(18.27) | death(Rrcurrence) | 80 | Male | IA | None |
| TCGA-55-8091* | 20(20) | alive | 74 | Male | IB | NA |
| TCGA-55-8097* | 15.87(15.87) | alive | 60 | Female | IA | NA |
| TCGA-55-8203* | 18.23(18.23) | alive | 69 | Female | IB | NA |
| TCGA-55-8204 | 17.17 | alive | 87 | Female | IB | UN |
| TCGA-55-8206 | 29.6 | alive | 56 | Male | IA | UN |
| TCGA-55-8207 | 32.57 | alive | 73 | Male | IB | UN |
| TCGA-55-8208* | 22.47(17.03) | alive(Rrcurrence) | 73 | Female | IA | UN |
| TCGA-55-8301 | 17.8 | alive | 58 | Male | IB | UN |
| TCGA-55-8302 | 15.93 | alive | 54 | Male | IB | UN |
| TCGA-55-8507 | 13.93 | alive | 53 | Male | IA | UN |
| TCGA-55-8510* | 17.97(17.97) | alive | 55 | Female | IB | NA |
| TCGA-55-8511 | 18.4 | alive | 73 | Female | IB | UN |
| TCGA-55-8514 | 17.33 | alive | 70 | Female | IB | UN |
| TCGA-55-8614* | 17.87(17.87) | alive | 76 | Male | IB | NA |
| TCGA-55-8616 | 1.6 | alive | 58 | Female | IB | UN |
| TCGA-55-8621* | 17.17(17.17) | alive | 75 | Female | IA | NA |
| TCGA-55-A491* | 20.87(20.87) | alive | 81 | Female | IA | NA |
| TCGA-55-A492* | 19.87(19.87) | alive | 70 | Female | IA | NA |
| TCGA-55-A493 | 0.93 | alive | 54 | Female | IB | UN |
| TCGA-55-A494* | 16.03(16.03) | alive | 61 | Female | IB | NA |
| TCGA-55-A4DG* | 20.27(20.27) | alive | 71 | Male | IA | NA |
| TCGA-55-A57B* | 18.2(18.2) | alive | 80 | Female | IA | NA |
| TCGA-62-A46O | 48.47 | death | 65 | Female | IB | UN |
| TCGA-62-A46P | 19.8 | death | 65 | Male | IB | UN |
| TCGA-62-A46R | 57.5 | death | 54 | Female | IB | UN |
| TCGA-62-A46V* | 73.3(73.3) | alive | 78 | Female | IB | NA |
| TCGA-62-A470 | 39.8 | death | 84 | Male | IB | UN |
| TCGA-64-1676* | 57.6(57.6) | alive | 58 | Male | IA | NA |
| TCGA-64-1681* | 38.9(14.63) | death(Rrcurrence) | 61 | Female | IA | YES |
| TCGA-67-3770 | 1.03 | alive | 70 | Female | IA | UN |
| TCGA-67-3771 | 20.33 | alive | 77 | Female | IA | UN |
| TCGA-67-3772 | 19.1 | alive | 82 | Female | IB | UN |
| TCGA-67-3773 | 14.23 | alive | 84 | Female | IB | UN |
| TCGA-67-3774 | 12.83 | alive | 73 | Female | IB | UN |
| TCGA-67-6216* | 4.7(4.7) | alive | 57 | Female | IA | NA |
| TCGA-69-7763* | 23(23) | alive | 69 | Male | IA | NA |
| TCGA-69-7764* | 13.8(13.8) | alive | 75 | Male | IA | NA |
| TCGA-69-7979 | 13.6 | alive | 71 | Female | IB | UN |
| TCGA-69-8255* | 4.3(4.3) | alive | 71 | Male | IA | NA |
| TCGA-71-6725 | 8.53 | alive | 48 | Female | IB | UN |
| TCGA-71-8520 | 7 | death | 60 | Female | IB | UN |
| TCGA-73-4658* | 53.33(53.33) | death | 80 | Female | IB | NA |
| TCGA-73-4662* | 83.83(0.7) | alive(Rrcurrence) | 65 | Female | IA | YES |
| TCGA-73-4677* | 1.27(1.27) | death | 74 | Male | IA | NA |
| TCGA-73-7499* | 51.03(51.03) | death | 81 | Female | IB | NA |
| TCGA-78-7143* | 165.37(50) | death(Rrcurrence) | 62 | Female | IB | None |
| TCGA-78-7153 | 121.17 | alive | 65 | Female | IB | NA |
| TCGA-78-7159 | 65.8 | alive | 60 | Female | IA | NA |
| TCGA-78-7163* | 241.6(241.6) | alive | 60 | Male | IB | None |
| TCGA-78-7537 | 54.07 | death | 72 | Male | IB | NA |
| TCGA-78-7540 | 39.9 | death | 66 | Female | IB | NA |
| TCGA-78-7542 | 10.7 | death | 56 | Male | IB | NA |
| TCGA-78-7633 | 50.93 | death | 67 | Male | IA | NA |
| TCGA-78-8655 | 78.67 | alive | 77 | Female | IA | NA |
| TCGA-78-8662 | 112.03 | death | 53 | Female | IB | UN |
| TCGA-83-5908* | 27.47(27.47) | alive | 59 | Female | IA | None |
| TCGA-86-7953* | 33.23(33.23) | alive | 69 | Female | IA | NA |
| TCGA-86-8073* | 24.67(24.67) | alive | 58 | Male | IB | NA |
| TCGA-86-8358* | 21.77(21.77) | alive | 44 | Male | IB | NA |
| TCGA-86-8585 | 11.77 | alive | 57 | Male | IB | UN |
| TCGA-86-8668* | 14.1(14.1) | alive | 61 | Female | IA | NA |
| TCGA-86-8673* | 28.73(28.73) | alive | 61 | Male | IB | NA |
| TCGA-86-A456* | 29.87(29.87) | alive | 78 | Female | IA | NA |
| TCGA-86-A4P7* | 13.83(13.83) | alive | 63 | Female | IB | NA |
| TCGA-91-6828* | 10.77(10.77) | alive | 70 | Male | IA | NA |
| TCGA-91-6829* | 41.93(41.93) | death | 78 | Male | IB | NA |
| TCGA-91-6831* | 10.33(10.33) | alive | 66 | Male | IB | NA |
| TCGA-91-6835* | 2.63(2.63) | alive | 81 | Female | IA | NA |
| TCGA-91-6836* | 13.9(13.9) | alive | 52 | Female | IB | NA |
| TCGA-91-6840* | 12.4(12.4) | alive | 59 | Female | IA | NA |
| TCGA-91-8496* | 16.83(16.83) | alive | 63 | Female | IB | NA |
| TCGA-91-8497* | 14.47(14.47) | death | 75 | Female | IA | NA |
| TCGA-91-8499* | 1.2(1.2) | alive | 76 | Female | IA | NA |
| TCGA-93-7347* | 22.77(22.77) | alive | 76 | Female | IA | NA |
| TCGA-93-7348* | 17.7(17.7) | alive | 75 | Female | IA | NA |
| TCGA-93-8067* | 6.2(6.2) | alive | 77 | Male | IB | NA |
| TCGA-93-A4JO | 1.1 | death | 70 | Male | IA | UN |
| TCGA-93-A4JQ* | 17.53(17.53) | alive | 49 | Male | IA | NA |
| TCGA-95-7043* | 16.77(16.77) | death | 63 | Female | IA | NA |
| TCGA-95-7944* | 12.57(12.57) | alive | 71 | Male | IA | NA |
| TCGA-95-7947* | 15.9(15.9) | alive | 67 | Male | IA | NA |
| TCGA-95-7948* | 15.87(15.87) | alive | 42 | Female | IB | NA |
| TCGA-95-8039* | 27.67(27.67) | alive | 72 | Male | IA | NA |
| TCGA-97-7546 | 42.83 | alive | 76 | Female | IA | UN |
| TCGA-97-7552* | 64.4(26.6) | alive(Rrcurrence) | 70 | Male | IB | None |
| TCGA-97-7553 | 62.33 | alive | 58 | Female | IA | UN |
| TCGA-97-7937 | 18.8 | alive | 65 | Male | IB | UN |
| TCGA-97-7938* | 0.6(0.6) | death | 76 | Female | IA | NA |
| TCGA-97-7941 | 16.13 | alive | 72 | Female | IA | UN |
| TCGA-97-8172* | 18.17(18.17) | alive | 75 | Female | IB | NA |
| TCGA-97-8179* | 14.5(14.5) | alive | 72 | Male | IA | NA |
| TCGA-97-A4LX* | 20.47(20.47) | alive | 81 | Male | IB | NA |
| TCGA-97-A4M0* | 21.73(21.73) | alive | 60 | Female | IB | NA |
| TCGA-97-A4M1* | 20.03(20.03) | alive | 52 | Female | IA | NA |
| TCGA-97-A4M2* | 20.8(20.8) | alive | 66 | Male | IA | NA |
| TCGA-97-A4M3* | 18(1.8) | alive(Rrcurrence) | 69 | Female | IA | None |
| TCGA-97-A4M5* | 21.13(21.13) | alive | 83 | Male | IA | NA |
| TCGA-97-A4M6* | 18.93(18.93) | alive | 45 | Female | IA | NA |
| TCGA-97-A4M7* | 20.97(20.97) | alive | 74 | Male | IA | NA |
| TCGA-99-8028* | 37.27(37.27) | alive | 50 | Female | IA | NA |
| TCGA-99-8032 | 1.47 | alive | 61 | Male | IA | UN |
| TCGA-99-AA5R* | 21.93(21.93) | alive | 70 | Female | IA | NA |
| TCGA-J2-A4AD* | 18.33(17.47) | death(Rrcurrence) | 61 | Female | IA | None |
| TCGA-J2-A4AE* | 35.97(35.97) | alive | 77 | Female | IA | NA |
| TCGA-J2-A4AG* | 32.93(32.93) | alive | 66 | Female | IA | NA |
| TCGA-L4-A4E6* | 14.5(14.5) | alive | 67 | Male | IA | NA |
| TCGA-L9-A443 | 6.43 | death | 63 | Female | IA | UN |
| TCGA-L9-A444 | 10.23 | alive | 60 | Female | IA | UN |
| TCGA-MN-A4N4* | 39.17(39.17) | alive | 57 | Male | IA | NA |
| TCGA-MN-A4N5* | 2.8(2.8) | alive | 63 | Male | IA | NA |
| TCGA-MP-A4SV | 87.33 | death | 67 | Male | IB | UN |
| TCGA-MP-A4TA | 31.67 | death | 75 | Female | IA | UN |
| TCGA-MP-A4TH* | 24.7(24.7) | alive | 70 | Female | IA | NA |
| TCGA-MP-A4TJ | 11.3 | death | 62 | Female | IA | UN |
| TCGA-MP-A5C7* | 74.93(74.93) | alive | 76 | Female | IB | NA |
| TCGA-NJ-A4YF* | 72.03(72.03) | alive | 50 | Female | IA | NA |
| TCGA-NJ-A4YG* | 63.47(63.47) | alive | 65 | Male | IB | NA |
| TCGA-NJ-A4YP* | 1.67(1.67) | alive | 52 | Male | IB | NA |
| TCGA-NJ-A4YQ* | 29.53(29.53) | alive | 69 | Female | IA | NA |
| TCGA-NJ-A55A* | 0.27(0.27) | alive | 76 | Female | IB | NA |
| TCGA-NJ-A55R* | 20.1(20.1) | alive | 67 | Male | IA | NA |
| TCGA-O1-A52J | 59.93 | death | 74 | Female | IA | UN |
| TCGA-S2-AA1A* | 17.1(17.1) | alive | 68 | Female | IA | NA |

Note: *represents the samples had recorded recurrence information. **^** the unit of OS and RFS is month.

**Table S2.** The 423 stage I-IV LUAD samples detected with multiple omic-data in TCGA.

| Sample id | stage | Sample id | stage | Sample id | stage |
| --- | --- | --- | --- | --- | --- |
| TCGA-05-4249 | I | TCGA-55-5899 | I | TCGA-78-7156 | IV |
| TCGA-05-4250 | III | TCGA-55-6543 | I | TCGA-78-7158 | III |
| TCGA-05-4382 | I | TCGA-55-6642 | I | TCGA-78-7160 | IV |
| TCGA-05-4384 | III | TCGA-55-6712 | II | TCGA-78-7161 | II |
| TCGA-05-4389 | I | TCGA-55-6968 | IV | TCGA-78-7162 | I |
| TCGA-05-4390 | I | TCGA-55-6969 | I | TCGA-78-7163 | I |
| TCGA-05-4396 | III | TCGA-55-6970 | III | TCGA-78-7166 | II |
| TCGA-05-4397 | II | TCGA-55-6971 | I | TCGA-78-7167 | IV |
| TCGA-05-4398 | III | TCGA-55-6972 | I | TCGA-78-7220 | III |
| TCGA-05-4402 | IV | TCGA-55-6975 | II | TCGA-78-7535 | I |
| TCGA-05-4403 | I | TCGA-55-6978 | II | TCGA-78-7536 | III |
| TCGA-05-4405 | I | TCGA-55-6979 | II | TCGA-78-7537 | I |
| TCGA-05-4415 | III | TCGA-55-6980 | I | TCGA-78-7539 | II |
| TCGA-05-4417 | I | TCGA-55-6981 | III | TCGA-78-7540 | I |
| TCGA-05-4418 | III | TCGA-55-6982 | II | TCGA-78-7542 | I |
| TCGA-05-4420 | I | TCGA-55-6983 | II | TCGA-78-7633 | I |
| TCGA-05-4422 | I | TCGA-55-6984 | II | TCGA-78-8640 | II |
| TCGA-05-4424 | II | TCGA-55-6985 | I | TCGA-78-8648 | II |
| TCGA-05-4426 | I | TCGA-55-6986 | I | TCGA-78-8660 | II |
| TCGA-05-4427 | II | TCGA-55-6987 | I | TCGA-86-6562 | II |
| TCGA-05-4430 | I | TCGA-55-7281 | I | TCGA-86-6851 | II |
| TCGA-05-4432 | II | TCGA-55-7283 | III | TCGA-86-7701 | IV |
| TCGA-05-4433 | I | TCGA-55-7284 | II | TCGA-86-7711 | II |
| TCGA-05-4434 | IV | TCGA-55-7570 | I | TCGA-86-7713 | II |
| TCGA-05-5420 | III | TCGA-55-7573 | I | TCGA-86-7714 | III |
| TCGA-05-5423 | II | TCGA-55-7574 | I | TCGA-86-7953 | I |
| TCGA-05-5425 | II | TCGA-55-7576 | I | TCGA-86-7954 | I |
| TCGA-05-5428 | II | TCGA-55-7724 | I | TCGA-86-7955 | I |
| TCGA-05-5429 | III | TCGA-55-7725 | I | TCGA-86-8054 | II |
| TCGA-05-5715 | I | TCGA-55-7726 | I | TCGA-86-8055 | II |
| TCGA-35-3615 | I | TCGA-55-7727 | III | TCGA-86-8056 | III |
| TCGA-35-4122 | I | TCGA-55-7728 | I | TCGA-86-8073 | I |
| TCGA-35-4123 | I | TCGA-55-7815 | I | TCGA-86-8074 | II |
| TCGA-35-5375 | III | TCGA-55-7903 | I | TCGA-86-8075 | I |
| TCGA-38-4625 | I | TCGA-55-7907 | II | TCGA-86-8076 | I |
| TCGA-38-4626 | II | TCGA-55-7910 | II | TCGA-86-8278 | II |
| TCGA-38-4627 | II | TCGA-55-7911 | I | TCGA-86-8279 | II |
| TCGA-38-4628 | II | TCGA-55-7914 | II | TCGA-86-8280 | II |
| TCGA-38-4629 | II | TCGA-55-7994 | II | TCGA-86-8358 | I |
| TCGA-38-4630 | I | TCGA-55-7995 | I | TCGA-86-8585 | I |
| TCGA-38-4631 | I | TCGA-55-8085 | I | TCGA-86-8668 | I |
| TCGA-38-4632 | IV | TCGA-55-8087 | I | TCGA-86-8669 | I |
| TCGA-38-6178 | III | TCGA-55-8089 | I | TCGA-86-8674 | II |
| TCGA-38-7271 | I | TCGA-55-8090 | I | TCGA-86-A456 | I |
| TCGA-38-A44F | I | TCGA-55-8091 | I | TCGA-86-A4D0 | II |
| TCGA-44-2655 | I | TCGA-55-8092 | II | TCGA-86-A4JF | II |
| TCGA-44-2656 | I | TCGA-55-8094 | IV | TCGA-86-A4P8 | III |
| TCGA-44-2659 | II | TCGA-55-8096 | I | TCGA-91-6828 | I |
| TCGA-44-2661 | I | TCGA-55-8097 | I | TCGA-91-6829 | I |
| TCGA-44-2662 | I | TCGA-55-8203 | I | TCGA-91-6830 | II |
| TCGA-44-2665 | II | TCGA-55-8204 | I | TCGA-91-6831 | I |
| TCGA-44-2666 | I | TCGA-55-8205 | II | TCGA-91-6835 | I |
| TCGA-44-2668 | I | TCGA-55-8206 | I | TCGA-91-6836 | I |
| TCGA-44-3396 | III | TCGA-55-8207 | I | TCGA-91-6840 | I |
| TCGA-44-3398 | I | TCGA-55-8208 | I | TCGA-91-6847 | I |
| TCGA-44-3918 | I | TCGA-55-8299 | I | TCGA-91-6848 | III |
| TCGA-44-3919 | I | TCGA-55-8301 | I | TCGA-91-6849 | III |
| TCGA-44-4112 | I | TCGA-55-8302 | I | TCGA-91-7771 | II |
| TCGA-44-5643 | III | TCGA-55-8505 | III | TCGA-91-8499 | I |
| TCGA-44-5644 | I | TCGA-55-8507 | I | TCGA-91-A4BC | II |
| TCGA-44-6145 | I | TCGA-55-8508 | II | TCGA-91-A4BD | II |
| TCGA-44-6146 | II | TCGA-55-8510 | I | TCGA-93-7347 | I |
| TCGA-44-6148 | I | TCGA-55-8511 | I | TCGA-93-7348 | I |
| TCGA-44-6774 | III | TCGA-55-8512 | IV | TCGA-93-8067 | I |
| TCGA-44-6775 | I | TCGA-55-8513 | II | TCGA-93-A4JN | IV |
| TCGA-44-6776 | I | TCGA-55-8614 | I | TCGA-93-A4JO | I |
| TCGA-44-6778 | I | TCGA-55-8615 | III | TCGA-93-A4JP | IV |
| TCGA-44-6779 | II | TCGA-55-8616 | I | TCGA-93-A4JQ | I |
| TCGA-44-7659 | I | TCGA-55-8619 | II | TCGA-95-7039 | II |
| TCGA-44-7660 | I | TCGA-55-8620 | IV | TCGA-95-7043 | I |
| TCGA-44-7661 | I | TCGA-55-A48X | II | TCGA-95-7562 | II |
| TCGA-44-7662 | I | TCGA-55-A48Y | II | TCGA-95-7567 | II |
| TCGA-44-7667 | II | TCGA-55-A48Z | III | TCGA-95-7944 | I |
| TCGA-44-7669 | II | TCGA-55-A490 | II | TCGA-95-7947 | I |
| TCGA-44-7670 | II | TCGA-55-A491 | I | TCGA-95-7948 | I |
| TCGA-44-7671 | I | TCGA-55-A492 | I | TCGA-95-8039 | I |
| TCGA-44-7672 | I | TCGA-55-A493 | I | TCGA-95-A4VK | III |
| TCGA-44-8117 | I | TCGA-55-A494 | I | TCGA-95-A4VN | II |
| TCGA-44-8119 | II | TCGA-55-A4DF | I | TCGA-95-A4VP | III |
| TCGA-44-8120 | I | TCGA-55-A4DG | I | TCGA-97-7546 | I |
| TCGA-44-A479 | I | TCGA-62-8395 | II | TCGA-97-7547 | I |
| TCGA-44-A47A | I | TCGA-62-A46O | I | TCGA-97-7552 | I |
| TCGA-44-A47B | I | TCGA-62-A46P | I | TCGA-97-7553 | I |
| TCGA-44-A47G | I | TCGA-62-A46R | I | TCGA-97-7554 | III |
| TCGA-44-A4SS | I | TCGA-62-A46S | I | TCGA-97-7937 | I |
| TCGA-44-A4SU | I | TCGA-62-A46U | II | TCGA-97-7938 | I |
| TCGA-49-4486 | I | TCGA-62-A46V | I | TCGA-97-7941 | I |
| TCGA-49-4487 | I | TCGA-62-A46Y | III | TCGA-97-8171 | IV |
| TCGA-49-4488 | I | TCGA-62-A470 | I | TCGA-97-8172 | I |
| TCGA-49-4490 | III | TCGA-62-A471 | II | TCGA-97-8174 | II |
| TCGA-49-4494 | III | TCGA-62-A472 | II | TCGA-97-8175 | I |
| TCGA-49-4501 | I | TCGA-64-1676 | I | TCGA-97-8176 | III |
| TCGA-49-4505 | II | TCGA-64-1677 | III | TCGA-97-8177 | I |
| TCGA-49-4506 | II | TCGA-64-1678 | II | TCGA-97-8179 | I |
| TCGA-49-4507 | III | TCGA-64-1679 | III | TCGA-97-A4LX | I |
| TCGA-49-4510 | II | TCGA-64-1680 | IV | TCGA-97-A4M1 | I |
| TCGA-49-4512 | III | TCGA-64-1681 | I | TCGA-97-A4M2 | I |
| TCGA-49-4514 | I | TCGA-64-5774 | I | TCGA-97-A4M3 | I |
| TCGA-49-6742 | II | TCGA-64-5778 | I | TCGA-97-A4M5 | I |
| TCGA-49-6743 | III | TCGA-64-5779 | III | TCGA-97-A4M6 | I |
| TCGA-49-6744 | II | TCGA-64-5781 | I | TCGA-97-A4M7 | I |
| TCGA-49-6745 | III | TCGA-64-5815 | II | TCGA-99-7458 | III |
| TCGA-49-6761 | III | TCGA-67-3770 | I | TCGA-99-8025 | III |
| TCGA-49-6767 | II | TCGA-67-3771 | I | TCGA-99-8028 | I |
| TCGA-50-5044 | III | TCGA-67-3772 | I | TCGA-99-8032 | I |
| TCGA-50-5045 | I | TCGA-67-3773 | I | TCGA-99-8033 | IV |
| TCGA-50-5049 | I | TCGA-67-3774 | I | TCGA-J2-8192 | II |
| TCGA-50-5051 | III | TCGA-67-6215 | I | TCGA-J2-8194 | II |
| TCGA-50-5055 | II | TCGA-67-6216 | I | TCGA-J2-A4AD | I |
| TCGA-50-5066 | I | TCGA-67-6217 | II | TCGA-J2-A4AE | I |
| TCGA-50-5068 | II | TCGA-69-7760 | II | TCGA-J2-A4AG | I |
| TCGA-50-5930 | III | TCGA-69-7761 | I | TCGA-L4-A4E6 | I |
| TCGA-50-5931 | I | TCGA-69-7763 | I | TCGA-L9-A443 | I |
| TCGA-50-5932 | II | TCGA-69-7764 | I | TCGA-L9-A444 | I |
| TCGA-50-5933 | III | TCGA-69-7765 | IV | TCGA-MN-A4N1 | II |
| TCGA-50-5935 | I | TCGA-69-7973 | I | TCGA-MN-A4N4 | I |
| TCGA-50-5936 | III | TCGA-69-7974 | III | TCGA-MN-A4N5 | I |
| TCGA-50-5939 | I | TCGA-69-7978 | II | TCGA-MP-A4SV | I |
| TCGA-50-5941 | III | TCGA-69-7979 | I | TCGA-MP-A4SW | II |
| TCGA-50-5942 | I | TCGA-69-8255 | I | TCGA-MP-A4SY | II |
| TCGA-50-5944 | I | TCGA-69-A59K | II | TCGA-MP-A4T4 | II |
| TCGA-50-5946 | I | TCGA-71-6725 | I | TCGA-MP-A4T6 | III |
| TCGA-50-6590 | I | TCGA-73-4659 | III | TCGA-MP-A4T7 | IV |
| TCGA-50-6591 | IV | TCGA-73-4662 | I | TCGA-MP-A4T8 | III |
| TCGA-50-6592 | I | TCGA-73-4666 | IV | TCGA-MP-A4T9 | III |
| TCGA-50-6593 | III | TCGA-73-4668 | II | TCGA-MP-A4TA | I |
| TCGA-50-6594 | III | TCGA-73-4670 | IV | TCGA-MP-A4TC | III |
| TCGA-50-6595 | III | TCGA-73-4675 | III | TCGA-MP-A4TD | III |
| TCGA-50-6597 | I | TCGA-73-4677 | I | TCGA-MP-A4TE | II |
| TCGA-50-7109 | I | TCGA-73-7498 | I | TCGA-MP-A4TH | I |
| TCGA-50-8457 | I | TCGA-73-7499 | I | TCGA-MP-A4TI | II |
| TCGA-50-8459 | II | TCGA-78-7145 | IV | TCGA-MP-A4TK | II |
| TCGA-50-8460 | I | TCGA-78-7146 | III | TCGA-MP-A5C7 | I |
| TCGA-53-7624 | IV | TCGA-78-7147 | II | TCGA-NJ-A4YF | I |
| TCGA-53-7626 | II | TCGA-78-7148 | II | TCGA-NJ-A4YG | I |
| TCGA-53-7813 | III | TCGA-78-7149 | III | TCGA-NJ-A4YI | III |
| TCGA-53-A4EZ | II | TCGA-78-7150 | II | TCGA-NJ-A4YP | I |
| TCGA-55-1592 | I | TCGA-78-7152 | I | TCGA-NJ-A4YQ | I |
| TCGA-55-1594 | III | TCGA-78-7153 | I | TCGA-NJ-A55A | I |
| TCGA-55-1595 | I | TCGA-78-7154 | III | TCGA-NJ-A55O | II |
| TCGA-55-1596 | II | TCGA-78-7155 | I | TCGA-O1-A52J | I |

**Table S3.** The functional pathways enriched with metastasis-related DE genes.

| **GO pathways** | **Path.G** | **M**.G | **p** |
| --- | --- | --- | --- |
| acute inflammatory response to antigenic stimulus | 21 | 14 | <0.0001 |
| anatomical structure formation involved in morphogenesis | 952 | 305 | <0.0001 |
| axoneme assembly | 52 | 33 | <0.0001 |
| behavior | 537 | 193 | <0.0001 |
| calcium ion homeostasis | 398 | 142 | <0.0001 |
| calcium ion transmembrane transport | 262 | 92 | <0.0001 |
| cAMP biosynthetic process | 110 | 52 | <0.0001 |
| cell adhesion | 1671 | 467 | <0.0001 |
| cell fate commitment | 242 | 85 | <0.0001 |
| cell maturation | 153 | 63 | <0.0001 |
| cell motility | 1311 | 399 | <0.0001 |
| cell proliferation | 1903 | 551 | <0.0001 |
| cell surface receptor signaling pathway | 2564 | 740 | <0.0001 |
| cell-cell signaling | 1510 | 472 | <0.0001 |
| chemical synaptic transmission | 601 | 202 | <0.0001 |
| chemokine-mediated signaling pathway | 81 | 38 | <0.0001 |
| cilium movement | 49 | 25 | <0.0001 |
| extracellular matrix organization | 322 | 116 | <0.0001 |
| glycerol transport | 13 | 10 | <0.0001 |
| G-protein coupled glutamate receptor signaling pathway | 13 | 10 | <0.0001 |
| G-protein coupled receptor signaling pathway, coupled to cyclic nucleotide second messenger | 190 | 88 | <0.0001 |
| homophilic cell adhesion via plasma membrane adhesion molecules | 158 | 65 | <0.0001 |
| import into cell | 72 | 35 | <0.0001 |
| inflammatory response | 644 | 213 | <0.0001 |
| negative regulation of cell differentiation | 603 | 200 | <0.0001 |
| nervous system development | 2106 | 631 | <0.0001 |
| neurotransmitter transport | 194 | 71 | <0.0001 |
| phospholipase C-activating G-protein coupled receptor signaling pathway | 111 | 46 | <0.0001 |
| positive regulation of inflammatory response | 114 | 51 | <0.0001 |
| potassium ion transmembrane transport | 182 | 80 | <0.0001 |
| proximal/distal pattern formation | 32 | 18 | <0.0001 |
| receptor-mediated endocytosis | 269 | 102 | <0.0001 |
| regulation of acute inflammatory response | 70 | 32 | <0.0001 |
| regulation of cellular component movement | 779 | 233 | <0.0001 |
| regulation of heart contraction | 230 | 97 | <0.0001 |
| regulation of hormone levels | 463 | 153 | <0.0001 |
| regulation of ion transmembrane transport | 383 | 143 | <0.0001 |
| regulation of membrane potential | 351 | 122 | <0.0001 |
| regulation of muscle contraction | 149 | 62 | <0.0001 |
| regulation of neurotransmitter levels | 193 | 71 | <0.0001 |
| regulation of secretion | 660 | 203 | <0.0001 |
| regulation of transmembrane transporter activity | 182 | 74 | <0.0001 |
| reproduction | 1314 | 388 | <0.0001 |
| response to gonadotropin | 27 | 16 | <0.0001 |
| response to lipid | 829 | 247 | <0.0001 |
| response to organic cyclic compound | 868 | 275 | <0.0001 |
| response to organophosphorus | 127 | 51 | <0.0001 |
| response to purine-containing compound | 141 | 56 | <0.0001 |
| secretion by cell | 936 | 276 | <0.0001 |
| sensory perception of pain | 91 | 40 | <0.0001 |
| smooth muscle contraction | 94 | 42 | <0.0001 |
| vasculature development | 595 | 182 | <0.0001 |
| glutamate receptor signaling pathway | 69 | 31 | 0.0001 |
| thyroid hormone metabolic process | 20 | 13 | 0.0001 |
| negative regulation of cell development | 270 | 91 | 0.0002 |
| embryonic skeletal system development | 121 | 47 | 0.0002 |
| neural retina development | 53 | 25 | 0.0002 |
| cAMP-mediated signaling | 56 | 26 | 0.0002 |
| branching involved in ureteric bud morphogenesis | 59 | 27 | 0.0002 |
| embryonic organ morphogenesis | 282 | 94 | 0.0002 |
| DNA replication-dependent nucleosome assembly | 31 | 17 | 0.0002 |
| mesenchymal cell differentiation | 185 | 66 | 0.0002 |
| anterior/posterior pattern specification | 199 | 70 | 0.0002 |
| regulation of fibrinolysis | 14 | 10 | 0.0002 |
| sensory perception of mechanical stimulus | 152 | 56 | 0.0002 |
| chondrocyte differentiation | 94 | 38 | 0.0003 |
| negative regulation of growth | 226 | 77 | 0.0003 |
| retina morphogenesis in camera-type eye | 46 | 22 | 0.0003 |
| detection of abiotic stimulus | 131 | 49 | 0.0004 |
| metanephros development | 89 | 36 | 0.0004 |
| regulation of interleukin-4 production | 27 | 15 | 0.0004 |
| DNA replication checkpoint | 17 | 11 | 0.0004 |
| CENP-A containing nucleosome assembly | 41 | 20 | 0.0005 |
| negative regulation of ion transport | 109 | 42 | 0.0005 |
| response to wounding | 631 | 187 | 0.0005 |
| acute-phase response | 47 | 22 | 0.0005 |
| response to glucocorticoid | 136 | 50 | 0.0005 |
| cellular response to sterol | 15 | 10 | 0.0005 |
| bone mineralization | 100 | 39 | 0.0006 |
| regulation of ion homeostasis | 195 | 67 | 0.0006 |
| DNA replication initiation | 39 | 19 | 0.0007 |
| cellular modified amino acid metabolic process | 154 | 55 | 0.0007 |
| glutamate secretion | 42 | 20 | 0.0007 |
| cellular water homeostasis | 13 | 9 | 0.0007 |
| regulation of systemic arterial blood pressure mediated by a chemical signal | 48 | 22 | 0.0007 |
| positive regulation of cell differentiation | 815 | 234 | 0.0007 |
| response to drug | 394 | 122 | 0.0008 |
| ferric iron import into cell | 5 | 5 | 0.0008 |
| negative regulation of skeletal muscle cell differentiation | 5 | 5 | 0.0008 |
| leukotriene biosynthetic process | 18 | 11 | 0.0008 |
| plasminogen activation | 18 | 11 | 0.0008 |
| odontogenesis | 115 | 43 | 0.0008 |
| positive regulation of T cell differentiation in thymus | 11 | 8 | 0.0009 |
| digestive tract development | 132 | 48 | 0.0009 |
| calcium-mediated signaling | 146 | 52 | 0.0010 |
| regulation of protein processing | 80 | 32 | 0.0010 |
| cGMP catabolic process | 7 | 6 | 0.0010 |
| elastic fiber assembly | 7 | 6 | 0.0010 |
| skeletal muscle satellite cell differentiation | 7 | 6 | 0.0010 |
| urea transmembrane transport | 7 | 6 | 0.0010 |
| dopamine metabolic process | 32 | 16 | 0.0012 |
| positive regulation of chemotaxis | 117 | 43 | 0.0013 |
| regulation of G-protein coupled receptor protein signaling pathway | 124 | 45 | 0.0013 |
| regulation of interferon-gamma production | 91 | 35 | 0.0014 |
| regulation of morphogenesis of a branching structure | 53 | 23 | 0.0014 |
| phagocytosis, recognition | 14 | 9 | 0.0015 |
| skeletal muscle organ development | 166 | 57 | 0.0016 |
| cAMP catabolic process | 19 | 11 | 0.0016 |
| positive regulation of protein phosphorylation | 872 | 246 | 0.0016 |
| complement activation, classical pathway | 30 | 15 | 0.0017 |
| response to calcium ion | 112 | 41 | 0.0018 |
| negative regulation of myoblast differentiation | 22 | 12 | 0.0019 |
| regulation of calcium ion-dependent exocytosis | 73 | 29 | 0.0020 |
| positive regulation of kidney development | 39 | 18 | 0.0020 |
| lactate transmembrane transport | 12 | 8 | 0.0021 |
| negative regulation of cardiocyte differentiation | 12 | 8 | 0.0021 |
| monoamine transport | 67 | 27 | 0.0022 |
| heat generation | 17 | 10 | 0.0022 |
| positive regulation of NF-kappaB import into nucleus | 25 | 13 | 0.0022 |
| excretion | 64 | 26 | 0.0023 |
| regulation of small GTPase mediated signal transduction | 294 | 92 | 0.0023 |
| dorsal/ventral pattern formation | 100 | 37 | 0.0023 |
| embryonic pattern specification | 58 | 24 | 0.0025 |
| chemokine production | 74 | 29 | 0.0025 |
| chemical homeostasis within a tissue | 10 | 7 | 0.0027 |
| negative regulation of pathway-restricted SMAD protein phosphorylation | 10 | 7 | 0.0027 |
| positive regulation of chemokine secretion | 10 | 7 | 0.0027 |
| respiratory gaseous exchange | 68 | 27 | 0.0028 |
| lung alveolus development | 40 | 18 | 0.0028 |
| negative chemotaxis | 40 | 18 | 0.0028 |
| positive regulation of lipase activity | 62 | 25 | 0.0031 |
| water transport | 23 | 12 | 0.0031 |
| developmental growth involved in morphogenesis | 210 | 68 | 0.0032 |
| cellular response to erythropoietin | 4 | 4 | 0.0033 |
| mesonephric duct morphogenesis | 4 | 4 | 0.0033 |
| pattern specification involved in metanephros development | 4 | 4 | 0.0033 |
| positive regulation of melanocyte differentiation | 4 | 4 | 0.0033 |
| positive regulation of ion transport | 203 | 66 | 0.0033 |
| sodium ion transmembrane transport | 136 | 47 | 0.0033 |
| fat cell differentiation | 200 | 65 | 0.0036 |
| muscle cell differentiation | 350 | 106 | 0.0036 |
| regulation of synapse organization | 116 | 41 | 0.0037 |
| collagen catabolic process | 66 | 26 | 0.0037 |
| positive regulation of interleukin-12 production | 32 | 15 | 0.0038 |
| cellular response to metal ion | 130 | 45 | 0.0038 |
| blood coagulation, intrinsic pathway | 18 | 10 | 0.0039 |
| protein heterotetramerization | 41 | 18 | 0.0039 |
| chromatin silencing at rDNA | 38 | 17 | 0.0039 |
| positive regulation of alpha-beta T cell differentiation | 38 | 17 | 0.0039 |
| hormone biosynthetic process | 63 | 25 | 0.0039 |

Path.G: Genes in the GO pathways; M.G, metastasis-related DE genes in the GO pathway.

**Table S4.** The genomic characteristics between the reclassified metastatic and nonmetastatic

groups with aid of 7/9-GPS.

|  | **Reclassified metastasis** | | **Reclassified non-metastasis** | |  |
| --- | --- | --- | --- | --- | --- |
| **Mutation/CNA** | **Alteration** | **Non-alteration** | **Alteration** | **Non-alteration** | **Fisher.p** |
| *TP53** | 201 | 114 | 22 | 86 | <0.0001 |
| *PTPRZ1* | 44 | 271 | 1 | 107 | <0.0001 |
| *CSMD3* | 147 | 168 | 23 | 85 | <0.0001 |
| *ABCB5* | 41 | 274 | 1 | 107 | <0.0001 |
| *DCDC5* | 30 | 285 | 0 | 108 | 0.0001 |
| *TCHH* | 36 | 279 | 1 | 107 | 0.0003 |
| *USH2A* | 125 | 190 | 22 | 86 | 0.0003 |
| *CNTN1* | 27 | 288 | 0 | 108 | 0.0004 |
| *SMARCA4* | 34 | 281 | 1 | 107 | 0.0004 |
| *NLRP3* | 42 | 273 | 2 | 106 | 0.0004 |
| *FLG2* | 62 | 253 | 6 | 102 | 0.0004 |
| *FER1L6* | 34 | 281 | 1 | 107 | 0.0004 |
| *TTN* | 172 | 143 | 38 | 70 | 0.0005 |
| *PDE3A* | 25 | 290 | 0 | 108 | 0.0007 |
| *SGIP1* | 26 | 289 | 0 | 108 | 0.0007 |
| *POM121L12* | 32 | 283 | 1 | 107 | 0.0007 |
| *CDH18* | 38 | 277 | 2 | 106 | 0.0009 |
| *RP1L1* | 71 | 244 | 9 | 99 | 0.0009 |
| *TG* | 48 | 267 | 4 | 104 | 0.001 |
| *SORCS1* | 48 | 267 | 4 | 104 | 0.001 |
| *ABCA6* | 31 | 284 | 1 | 107 | 0.0012 |
| 3q26.2 (amp) | 76 | 239 | 6 | 102 | <0.0001 |
| 7q31.2(amp) | 92 | 223 | 9 | 99 | <0.0001 |
| 3p26.3(del) | 85 | 230 | 7 | 101 | <0.0001 |
| 3p21.31(del) | 93 | 222 | 10 | 98 | <0.0001 |
| 17p11.2(del) | 102 | 213 | 14 | 94 | <0.0001 |
| 5q35.1(amp) | 32 | 283 | 28 | 80 | 0.0002 |
| 7p11.2(amp) | 124 | 191 | 21 | 87 | 0.0002 |
| 11q13.3(amp) | 72 | 243 | 8 | 100 | 0.0002 |
| 19q12(amp) | 65 | 250 | 7 | 101 | 0.0005 |
| 17p13.3(del) | 117 | 198 | 21 | 87 | 0.0008 |
| 22q13.32(del) | 86 | 229 | 13 | 95 | 0.0009 |
| 7p21.1(amp) | 131 | 184 | 26 | 82 | 0.0012 |
| 5q31.1(del) | 84 | 231 | 13 | 95 | 0.0014 |
| 18q21.32(del) | 105 | 210 | 19 | 89 | 0.0021 |
| 15q11.2(del) | 94 | 221 | 16 | 92 | 0.0022 |
| 8q11.1(amp) | 108 | 207 | 20 | 88 | 0.0023 |
| 12p12.1(amp) | 78 | 237 | 12 | 96 | 0.0026 |
| 8p11.23(amp) | 61 | 254 | 8 | 100 | 0.0038 |
| 18q23(del) | 104 | 211 | 20 | 88 | 0.0047 |
| 9q21.11(del) | 95 | 220 | 18 | 90 | 0.0057 |
| 12q14.1(amp) | 64 | 251 | 10 | 98 | 0.0082 |
| 12q15(amp) | 64 | 251 | 10 | 98 | 0.0082 |
| 13q14.2(del) | 100 | 215 | 20 | 88 | 0.0092 |
| 5q11.2(del) | 70 | 245 | 12 | 96 | 0.0111 |
| 20p13(del) | 58 | 257 | 9 | 99 | 0.014 |
| 3q29(del) | 37 | 278 | 23 | 85 | 0.0169 |
| 8p23.2(del) | 116 | 199 | 26 | 82 | 0.018 |

**Table S5.** The genomic characteristics between the stage I high-risk and low-risk samples identified by 7/9-GPS.

|  | **Reclassified metastasis** | | **Reclassified non-metastasis** | |  |
| --- | --- | --- | --- | --- | --- |
| **Mutation/CNA** | **Alteration** | **Non-alteration** | **Alteration** | **Non-alteration** | **Fisher.p** |
| *TP53** | 96 | 41 | 19 | 72 | <0.0001 |
| *PTPRZ1* | 22 | 115 | 1 | 90 | <0.0001 |
| *CSMD3* | 72 | 65 | 19 | 72 | <0.0001 |
| *ABCB5* | 17 | 120 | 1 | 90 | 0.0018 |
| *DCDC5* | 12 | 125 | 0 | 91 | 0.002 |
| *TCHH* | 23 | 114 | 1 | 90 | <0.0001 |
| *USH2A* | 67 | 70 | 18 | 73 | <0.0001 |
| *CNTN1* | 15 | 122 | 0 | 91 | 0.0005 |
| *SMARCA4* | 13 | 124 | 1 | 90 | 0.0098 |
| *NLRP3* | 26 | 111 | 2 | 89 | <0.0001 |
| *FLG2* | 28 | 109 | 5 | 86 | 0.0018 |
| *FER1L6* | 17 | 120 | 1 | 90 | 0.0018 |
| *TTN* | 79 | 58 | 33 | 58 | 0.0019 |
| *PDE3A* | 11 | 126 | 0 | 91 | 0.0037 |
| *SGIP1* | 12 | 125 | 0 | 91 | 0.002 |
| *POM121L12* | 17 | 120 | 0 | 91 | 0.0001 |
| *CDH18* | 19 | 118 | 2 | 89 | 0.0021 |
| *RP1L1* | 32 | 105 | 7 | 84 | 0.0021 |
| *TG* | 23 | 114 | 3 | 88 | 0.0013 |
| *SORCS1* | 29 | 108 | 3 | 88 | <0.0001 |
| *ABCA6* | 16 | 121 | 1 | 90 | 0.0033 |
| 3q26.2 (amp) | 39 | 98 | 3 | 88 | <0.0001 |
| 7q31.2(amp) | 41 | 96 | 7 | 84 | <0.0001 |
| 3p26.3(del) | 36 | 101 | 7 | 84 | 0.0004 |
| 3p21.31(del) | 41 | 96 | 9 | 82 | 0.0003 |
| 17p11.2(del) | 45 | 92 | 14 | 77 | 0.0033 |
| 5q35.1(amp) | 12 | 125 | 27 | 64 | <0.0001 |
| 7p11.2(amp) | 51 | 86 | 17 | 74 | 0.003 |
| 11q13.3(amp) | 29 | 108 | 6 | 85 | 0.0026 |
| 19q12(amp) | 30 | 107 | 7 | 84 | 0.0054 |
| 17p13.3(del) | 55 | 82 | 20 | 71 | 0.006 |
| 22q13.32(del) | 36 | 101 | 12 | 79 | 0.0202 |
| 7p21.1(amp) | 55 | 82 | 22 | 69 | 0.015 |
| 5q31.1(del) | 41 | 96 | 9 | 82 | 0.0003 |
| 18q21.32(del) | 38 | 99 | 14 | 77 | 0.0359 |
| 15q11.2(del) | 42 | 95 | 14 | 77 | 0.0115 |
| 8q11.1(amp) | 52 | 85 | 18 | 73 | 0.0035 |
| 12p12.1(amp) | 37 | 100 | 6 | 85 | 0.0001 |
| 8p11.23(amp) | 35 | 102 | 8 | 83 | 0.0017 |
| 18q23(del) | 37 | 100 | 16 | 75 | 0.1111 |
| 9q21.11(del) | 46 | 91 | 15 | 76 | 0.0057 |
| 12q14.1(amp) | 28 | 109 | 6 | 85 | 0.0041 |
| 12q15(amp) | 28 | 109 | 6 | 85 | 0.0041 |
| 13q14.2(del) | 45 | 92 | 16 | 75 | 0.0142 |
| 5q11.2(del) | 33 | 104 | 9 | 82 | 0.0084 |
| 20p13(del) | 21 | 116 | 9 | 82 | 0.3174 |
| 3q29(del) | 14 | 123 | 18 | 73 | 0.0517 |
| 8p23.2(del) | 56 | 81 | 22 | 69 | 0.0104 |

Note: 45 of the 48 genomic lesions characterizing the difference between the reclassified metastatic samples and non-metastatic samples also had significantly different mutation or CNA frequencies between the stage I high-risk and low-risk samples identified by 7/9-GPS.

**Table S6.** The functional pathways enriched with differentially expressed genes regulated by each "driver" for reclassified metastatic samples.

| **ATRIP**  DNA replication//DNA replication checkpoint//double-strand break repair via homologous recombination//double-strand break repair via break-induced replication//DNA unwinding involved in DNA replication//DNA replication initiation//mitotic cell cycle phase transition//regulation of cell cycle process//G2 DNA damage checkpoint//interstrand cross-link repair//protein K6-linked ubiquitination//regulation of histone H3-K9 acetylation |
| --- |
| **CACNA2D2**  MAPK cascade//muscle contraction//ion transport//glutamate receptor signaling pathway//synaptic ve  sicle maturation//muscle filament sliding//regulation of muscle filament sliding speed//angiotensin-  activated signaling pathway//regulation of ATPase activity//positive regulation of Ras protein signa  l transduction//regulation of synaptic plasticity//cardiac muscle tissue development//neuromuscular  process//positive regulation of hydrolase activity//striated muscle cell development//heart contract  ion |
| **CATSPER3**  MAPK cascade//muscle contraction//calcium ion transport//glutamate receptor signaling pathway//memory//response to organonitrogen compound//muscle filament sliding//myofibril assembly//positive regulation of GTPase activity//angiotensin-activated signaling pathway//regulation of membrane potential//positive regulation of Ras protein signal transduction//regulation of neuronal synaptic plasticity//cell development//neuromuscular process//excitatory postsynaptic potential |
| **CCND1**  //DNA damage checkpoint//transcription from RNA polymerase II promoter//regulation of cell cycle//mi  totic cell cycle phase transition//cell proliferation//negative regulation of biosynthetic process//  DNA damage response, signal transduction by p53 class mediator//Leydig cell differentiation//mammary  gland alveolus development//regulation of cell cycle arrest//lens fiber cell apoptotic process |
| **CCNE1**  //DNA replication//DNA synthesis involved in DNA repair//strand displacement//regulation of cell cyc  le//double-strand break repair//DNA damage response, signal transduction by p53 class mediator resul  ting in transcription of p21 class mediator//intrinsic apoptotic signaling pathway in response to DN  A damage//positive regulation of transcription, DNA-templated//cell cycle process//chromosome breaka  ge//intrinsic apoptotic signaling pathway by p53 class mediator//protein K6-linked ubiquitination//l  ens fiber cell apoptotic process |
| **CDK4**  //regulation of cell cycle//mitotic cell cycle phase transition//regulation of cell cycle arrest//le  ns fiber cell apoptotic process |
| **CPSF6**  //positive regulation of inflammatory response to antigenic stimulus//positive regulation of immunog  lobulin mediated immune response//transmembrane receptor protein tyrosine kinase signaling pathway//positive regulation of phospholipase C activity//regulation of phosphatidylinositol 3-kinase signaling//peptidyl-tyrosine phosphorylation//interleukin-4 production//Fc-epsilon receptor signaling pathway//regulation of cell proliferation//phosphatidylinositol phosphorylation//antigen receptor-mediated signaling pathway//positive regulation of cell activation//regulation of lymphocyte activation |
| **CSNK2A1**  cell adhesion//homophilic cell adhesion via plasma membrane adhesion molecules//cell-cell adhesion//cell-cell adhesion via plasma-membrane adhesion molecules |
| **EGFR**  //cytokine production//retina homeostasis//adaptive immune response//positive regulation of inflamma  tory response to antigenic stimulus//transmembrane receptor protein tyrosine kinase signaling pathwa  y//intracellular signal transduction//homophilic cell adhesion via plasma membrane adhesion molecule  s//calcium-mediated signaling//positive regulation of cell proliferation//regulation of phosphatidyl  inositol 3-kinase signaling//single organismal cell-cell adhesion//T cell receptor signaling pathway  //lymphocyte differentiation//interleukin-4 production//adherens junction organization//Fc-epsilon r  eceptor signaling pathway//B cell activation//inositol phosphate metabolic process//positive regulat  ion of interleukin-2 biosynthetic process//phosphatidylinositol phosphorylation//B cell receptor sig  naling pathway//positive regulation of cell activation//regulation of lymphocyte activation//regulat  ion of phospholipase C activity |
| **FRS2**  //organ induction//adaptive immune response//positive regulation of inflammatory response to antigen  ic stimulus//apoptotic process//transmembrane receptor protein tyrosine kinase signaling pathway//in  tracellular signal transduction//embryonic pattern specification//fibroblast growth factor receptor  signaling pathway//positive regulation of phospholipase C activity//regulation of cell death//growth  factor dependent regulation of skeletal muscle satellite cell proliferation//peptidyl-tyrosine phos  phorylation//T cell receptor signaling pathway//lymphocyte differentiation//positive regulation of v  ascular endothelial growth factor receptor signaling pathway//embryonic camera-type eye development/  /T cell costimulation//interleukin-4 production//somatic stem cell population maintenance//phosphati  dylinositol-3-phosphate biosynthetic process//Fc-epsilon receptor signaling pathway//wound healing//  positive regulation of interleukin-2 biosynthetic process//negative regulation of insulin receptor s  ignaling pathway//alpha-beta T cell activation//phosphatidylinositol phosphorylation//positive regul  ation of lymphocyte proliferation//positive regulation of urothelial cell proliferation//regulation  of epithelial cell proliferation//regulation of developmental process//B cell receptor signaling pat  hway//positive regulation of B cell activation//positive chemotaxis//positive regulation of DNA meta  bolic process//positive regulation of NF-kappaB transcription factor activity//bronchiole morphogene  sis//mesenchymal-epithelial cell signaling involved in lung development//prostate gland epithelium m  orphogenesis//semicircular canal fusion//lung proximal/distal axis specification//morphogenesis of a  branching epithelium//regulation of lymphocyte apoptotic process//positive regulation of white fat  cell proliferation |
| **GNAI2**  //cell-cell signaling//renal water homeostasis//intracellular signal transduction//protein phosphory  lation//calcium ion transport//phagocytosis//response to stress//adenylate cyclase-inhibiting G-prot  ein coupled receptor signaling pathway//Ras protein signal transduction//regulation of insulin secre  tion//lipid catabolic process//second-messenger-mediated signaling//platelet activation//positive re  gulation of GTPase activity//Fc receptor signaling pathway//inositol phosphate metabolic process//le  ukocyte activation//regulation of adenylate cyclase activity//chemical homeostasis//antigen receptor  -mediated signaling pathway//B cell receptor signaling pathway//regulation of vesicle-mediated trans  port//cellular response to organonitrogen compound//regulation of phospholipase C activity |
| **IL13**  //regulation of immunoglobulin production//mast cell degranulation |
| **IL31RA**  positive regulation of tyrosine phosphorylation of Stat3 protein |
| **IL6ST**  //leukocyte homeostasis//positive regulation of cytokine production//CD8-positive, alpha-beta T cell  differentiation involved in immune response//leukocyte activation involved in immune response//posi  tive regulation of adaptive immune response//negative regulation of apoptotic process//regulation of  gliogenesis//neutrophil chemotaxis//interleukin-17 production//interleukin-11-mediated signaling pa  thway//T cell proliferation//positive regulation of tyrosine phosphorylation of STAT protein//positi  ve regulation of regulatory T cell differentiation//negative regulation of heart contraction//positi  ve regulation of isotype switching to IgG isotypes//positive regulation of inflammatory response//po  sitive regulation of neurogenesis//positive regulation of fructose 1,6-bisphosphate 1-phosphatase ac  tivity//positive regulation of fructose 1,6-bisphosphate metabolic process//interleukin-27-mediated  signaling pathway//positive regulation of tumor necrosis factor (ligand) superfamily member 11 produ  ction |
| **IRF1**  //microtubule cytoskeleton organization//DNA synthesis involved in DNA repair//strand displacement//  cell morphogenesis//angiogenesis//cell growth//neuron migration//positive regulation of cytokine pro  duction//startle response//norepinephrine-epinephrine vasoconstriction involved in regulation of sys  temic arterial blood pressure//positive regulation of heart rate by epinephrine-norepinephrine//adap  tive immune response//cytokine production involved in immune response//leukocyte mediated immunity//MyD88-dependent toll-like receptor signaling pathway//cell-cell signaling//regulation of systemic arterial blood pressure mediated by a chemical signal//actin cytoskeleton organization//apoptotic process//transmembrane receptor protein tyrosine kinase signaling pathway//intracellular signal transduction//protein complex assembly//protein phosphorylation//calcium ion transport//smooth muscle contraction//inflammatory response//centrosome cycle//cell-matrix adhesion//transmembrane receptor protein  tyrosine phosphatase signaling pathway//adenylate cyclase-modulating G-protein coupled receptor sig  naling pathway//phospholipase C-activating G-protein coupled receptor signaling pathway//positive re  gulation of cytosolic calcium ion concentration//integrin-mediated signaling pathway//calcium-mediat  ed signaling//embryo development//synapse assembly//central nervous system development//neuromuscular junction development//aging//excretion//learning or memory1//cell proliferation//attachment of spindle microtubules to kinetochore//response to wounding//positive regulation of signal transduction//specification of animal organ identity//response to organonitrogen compound//regulation of cell fatecommitment//positive regulation of phospholipase activity//positive regulation of gene expression//negative regulation of epithelial cell migration//negative regulation of cell development//regulation of fibroblast migration//positive regulation of glucose transport//telomere maintenance via telomere lengthening//negative regulation of cell cycle process//positive regulation of neuron projection development//regulation of gliogenesis//regulation of phosphatidylinositol 3-kinase signaling//response to organic cyclic compound//cytokine-mediated signaling pathway//taurine metabolic process//gland morphogenesis//T cell receptor signaling pathway//negative regulation of signaling//hemopoiesis//regulation of endocytosis//regulation of cell adhesion//platelet activation//bone mineralization//positive regulation of cell migration//mammary gland development//chromosome breakage//animal organ regeneration//positive regulation of cellular metabolic process//positive regulation of protein ubiquitination//actin cytoskeleton reorganization//spindle checkpoint//receptor internalization//regulation  of neurological system process//regulation of cytokinesis//response to lipopolysaccharide//interleu  kin-4 production//regulation of interleukin-6 production//positive regulation of interleukin-10 prod  uction//positive regulation of interleukin-12 production//cellular response to hormone stimulus//ino  sitol phosphate biosynthetic process//regulation of GTPase activity//protein localization to kinetoc  hore//organ growth//peptidyl-tyrosine dephosphorylation//Fc-epsilon receptor signaling pathway//neur  otrophin signaling pathway//locomotion//epithelial fluid transport//cytokine biosynthetic process//r  egulation of protein catabolic process//positive regulation of cell fate specification//positive reg  ulation of programmed cell death//negative regulation of programmed cell death//regulation of protei  n binding//positive regulation of blood vessel endothelial cell migration//leukocyte activation//bon  e resorption//positive regulation of cell differentiation//positive regulation of osteoblast differe  ntiation//negative regulation of heart contraction//positive regulation of lipid metabolic process//  regulation of glucose import//phosphatidylinositol phosphorylation//Tie signaling pathway//positive  regulation of developmental growth//regulation of behavior//B cell receptor signaling pathway//posit  ive regulation of cell activation//leukocyte migration//positive chemotaxis//regulation of secretion  //negative regulation of transport//positive regulation of DNA metabolic process//positive regulatio  n of NF-kappaB transcription factor activity//positive regulation of transferase activity//negative  regulation of oxidoreductase activity//organelle localization//positive regulation of cell division/  /regulation of protein kinase B signaling//regulation of cardiac muscle contraction//transdifferenti  ation//regulation of sarcomere organization//cell chemotaxis//regulation of morphogenesis of a branc  hing structure//regulation of dendritic spine development//heart trabecula morphogenesis//regulation  of establishment of protein localization//leukocyte aggregation//regulation of microtubule cytoskel  eton organization//cellular response to inorganic substance//cellular response to manganese ion//cel  lular response to growth factor stimulus//cellular response to lipid//signal transduction by p53 cla  ss mediator//protein K6-linked ubiquitination//positive regulation of cell cycle process//response t  o alcohol//positive regulation of protein localization to nucleus//regulation of organelle assembly/  /positive regulation of intracellular signal transduction//regulation of protein localization to pla  sma membrane//negative regulation of oxidative stress-induced neuron death//negative regulation of h  ydrogen peroxide-induced cell death//regulation of cell maturation//regulation of retrograde transpo  rt, endosome to Golgi//positive regulation of protein localization to membrane//mitotic recombinatio  n-dependent replication fork processing//regulation of ion homeostasis//positive regulation of cardi  ac muscle cell differentiation |
| **KIF3A**  //antigen processing and presentation of exogenous peptide antigen via MHC class II//T cell receptor  signaling pathway//T cell costimulation//interferon-gamma-mediated signaling pathway |
| **KRAS**  //Ras protein signal transduction//positive regulation of cellular senescence |
| **MAPK7**  //positive regulation of transcription from RNA polymerase II promoter//cellular response to fluid s  hear stress//cellular response to transforming growth factor beta stimulus |
| **MDM2**  //exit from mitosis//free ubiquitin chain polymerization//protein K29-linked ubiquitination//protein  K27-linked ubiquitination//protein K63-linked ubiquitination//protein K11-linked ubiquitination//pr  otein K6-linked ubiquitination//positive regulation of cell cycle process//regulation of mitotic cel  l cycle phase transition |
| **MED4**  //positive regulation of macrophage derived foam cell differentiation//positive regulation of choles  terol storage//triglyceride catabolic process//white fat cell differentiation//brown fat cell differ  entiation//lipid homeostasis//cellular response to lithium ion |
| **MED9**  //positive regulation of macrophage derived foam cell differentiation//positive regulation of choles  terol storage//triglyceride catabolic process//white fat cell differentiation//lipid homeostasis//ce  llular response to lithium ion |
| **PAFAH1B1**  centrosome localization |
| **PIP5K1B**  //organ induction//cytokine production//positive regulation of receptor internalization//positive re  gulation of inflammatory response to antigenic stimulus//positive regulation of immunoglobulin media  ted immune response//axis elongation//transmembrane receptor protein tyrosine kinase signaling pathway//phosphatidylinositol biosynthetic process//positive regulation of phospholipase activity//regulation of cell death//regulation of phosphatidylinositol 3-kinase signaling//single organismal cell-cell adhesion//peptidyl-tyrosine phosphorylation//lymphocyte differentiation//interleukin-4 production//inositol phosphate biosynthetic process//organg rowth//Fc-epsilon receptor signaling pathway//Fc-gamma receptor signaling pathway involved in phagocytosis//positive regulation of cell fate specification//muscle cell fate commitment//positive regulation of phosphatidylinositol 3-kinase activity//phosphatidylinositol phosphorylation//negative regulation of developmental growth//collateral sprouting//positive regulation of lymphocyte proliferation//positive regulation of epithelial cell proliferation//antigen receptor-mediated signaling pathway//positive regulation of B cell activation//regulation of chemotaxis//positive regulation of hydrolase activity//positive regulation of cardiac muscle tissue development//positive regulation of ERK1 and ERK2 cascade//apoptotic signaling pathway |
| **PLK2**  //DNA damage response, signal transduction by p53 class mediator resulting in cell cycle arrest//reg  ulation of centriole replication |
| **PMAIP1**  //release of cytochrome c from mitochondria//reactive oxygen species metabolic process//extrinsic ap  optotic signaling pathway via death domain receptors//intrinsic apoptotic signaling pathway in respo  nse to DNA damage//response to UV//response to ionizing radiation//negative regulation of cellular p  H reduction//cellular response to glucose starvation//T cell homeostasis//CD8-positive, alpha-beta T  cell lineage commitment//regulation of neuron apoptotic process//negative regulation of retinal cel  l programmed cell death//defense response to virus//regulation of mitochondrial membrane potential//  intrinsic apoptotic signaling pathway in response to endoplasmic reticulum stress//cellular response  to hypoxia//positive regulation of protein insertion into mitochondrial membrane involved in apopto  tic signaling pathway//regulation of extrinsic apoptotic signaling pathway//positive regulation of i  ntrinsic apoptotic signaling pathway |
| **PPP2CA**  /DNA damage induced protein phosphorylation//DNA damage response, signal transduction by p53 class  mediator resulting in transcription of p21 class mediator//cell aging//replicative senescence//mitot  ic cell cycle process |
| **RB1**  //G1/S transition of mitotic cell cycle//regulation of cell cycle//negative regulation of cell proli  feration//positive regulation of transcription from RNA polymerase II promoter//positive regulation  of binding//transdifferentiation//lens fiber cell apoptotic process |
| **RHOA**  //G1/S transition of mitotic cell cycle//positive regulation of protein phosphorylation//actin filam  ent-based process//cell division//cardiac muscle cell development//positive regulation of cell cycle  process |
| **RILP**  //antigen processing and presentation of exogenous peptide antigen via MHC class II//T cell receptor  signaling pathway//T cell costimulation//interferon-gamma-mediated signaling pathway |
| **RPA1**  //chromosome organization//G1/S transition of mitotic cell cycle//telomere maintenance via recombina  tion//DNA synthesis involved in DNA repair//DNA-dependent DNA replication//interstrand cross-link re  pair |
| **SKP1**  //DNA replication//regulation of cell cycle//mitotic cell cycle phase transition |
| **TP53**  chromosome organization//microtubule cytoskeleton organization//DNA strand renaturation//cell grow  th//release of cytochrome c from mitochondria//regulation of protein phosphorylation//DNA metabolic  process//protein complex assembly//DNA damage response, signal transduction by p53 class mediator resulting in cell cycle arrest//mitotic nuclear division//cell surface receptor signaling pathway//agi  ng//positive regulation of cell proliferation//negative regulation of cell proliferation//response t  o metal ion//response to external stimulus//positive regulation of signal transduction//response to  X-ray//response to organonitrogen compound//proteasomal protein catabolic process//regulation of glu  cose metabolic process//response to organic cyclic compound//regulation of apoptotic process//cell m  igration//peptidyl-serine phosphorylation//peptidyl-tyrosine phosphorylation//positive regulation of  cellular metabolic process//negative regulation of protein ubiquitination//regulation of organelle  organization//regulation of growth//tissue regeneration//positive regulation of phosphorylation//res  ponse to hydrogen peroxide//positive regulation of insulin-like growth factor receptor signaling pat  hway//single organism reproductive process//positive regulation of cell differentiation//ephrin rece  ptor signaling pathway//response to axon injury//anatomical structure development//regulation of cat  alytic activity//positive regulation of transport//ventricular cardiac muscle cell development//regu  lation of cellular localization//mammary gland morphogenesis//mammary gland epithelium development//regulation of establishment of protein localization//thymocyte apoptotic process//aggresome assembly//cellular response to inorganic substance//cellular response to hypoxia//mitotic cell cycle arrest//replicative senescence//fasciculation of sensory neuron axon//fasciculation of motor neuron axon//regulation of mitotic cell cycle phase transition//regulation of cell cycle G2/M phase transition//positive regulation of oxidative stress-induced neuron death//positive regulation of protein ubiquitination involved in ubiquitin-dependent protein catabolic process |
| **TPCN2**  //MAPK cascade//startle response//muscle contraction//calcium ion transport//muscle filament sliding  //myofibril assembly//ionotropic glutamate receptor signaling pathway//angiotensin-activated signali  ng pathway//regulation of ATPase activity//positive regulation of Ras protein signal transduction//r  egulation of neuronal synaptic plasticity//cardiac muscle tissue development//positive regulation of  hydrolase activity |
| **TREX1**  //DNA replication//DNA synthesis involved in DNA repair//inner cell mass cell proliferation//double-  strand break repair//DNA recombination//DNA damage induced protein phosphorylation//DNA damage response, signal transduction by p53 class mediator resulting in transcription of p21 class mediator//reg  ulation of mitotic cell cycle//cell aging//intrinsic apoptotic signaling pathway in response to DNA  damage//response to ionizing radiation//negative regulation of cell cycle process//positive regulati  on of transcription, DNA-templated//chromosome breakage//G2 DNA damage checkpoint//histone H3 acetylation//histone H4 acetylation//regulation of centrosome cycle//centrosome duplication//positive regulation of histone H4-K20 methylation//positive regulation of cell cycle arrest//replicative senescen  ce//regulation of signal transduction by p53 class mediator//cellular response to bisphenol A//regul  ation of response to DNA damage stimulus |
| **TRPV2**  //muscle contraction//muscle filament sliding//myofibril assembly//angiotensin-activated signaling p  athway |
| **UBA7**  //exit from mitosis//free ubiquitin chain polymerization//protein K29-linked ubiquitination//protein  K27-linked ubiquitination//protein K63-linked ubiquitination//protein K11-linked ubiquitination//pr  otein K6-linked ubiquitination |
| **UBE3A**  //exit from mitosis//free ubiquitin chain polymerization//positive regulation of protein ubiquitinat  ion//protein K29-linked ubiquitination//protein K27-linked ubiquitination//protein K48-linked ubiqui  tination//protein K11-linked ubiquitination//protein K6-linked ubiquitination |
| **YWHAE**  //DNA replication//DNA replication checkpoint//G2/M transition of mitotic cell cycle//regulation of  mitotic nuclear division//mitotic cell cycle checkpoint//mitotic centrosome separation//negative reg  ulation of cell cycle process//histone phosphorylation//G2 DNA damage checkpoint//regulation of cent  rosome cycle//signal transduction by p53 class mediator |


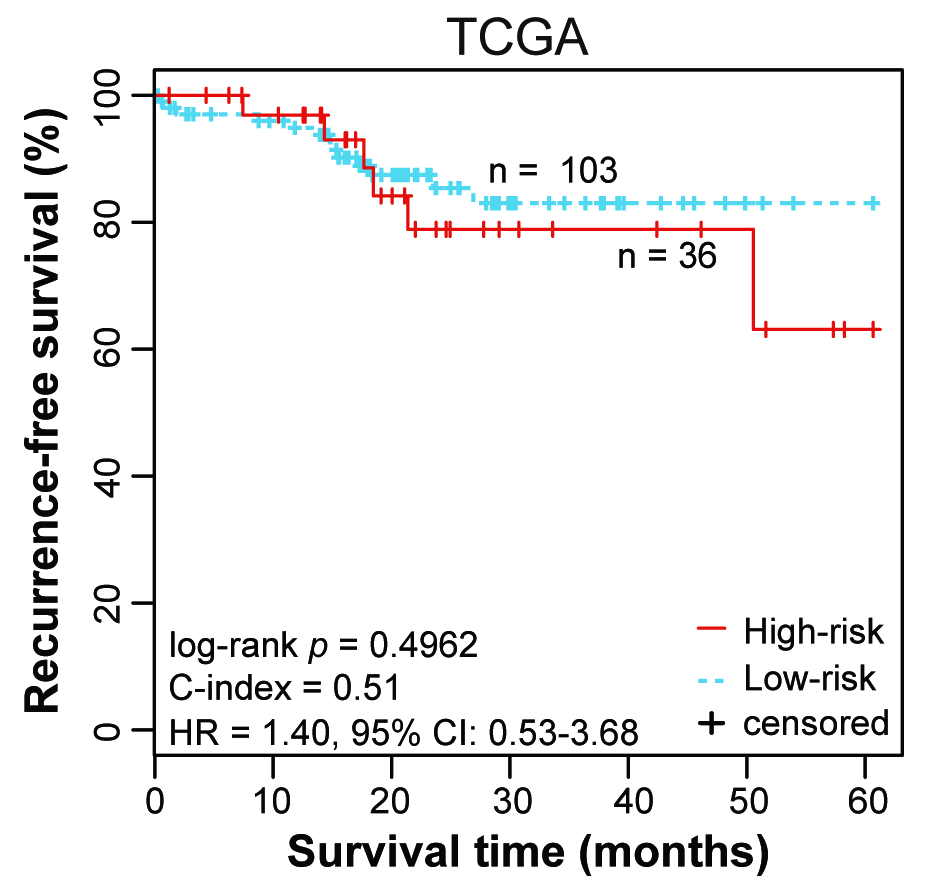


**Figure S1—The Kaplan–Meier curves of recurrence-free survival (****RFS) for 139 stage I LUAD samples stratified by 9-GPS based on the majority voting rule in TCGA.**


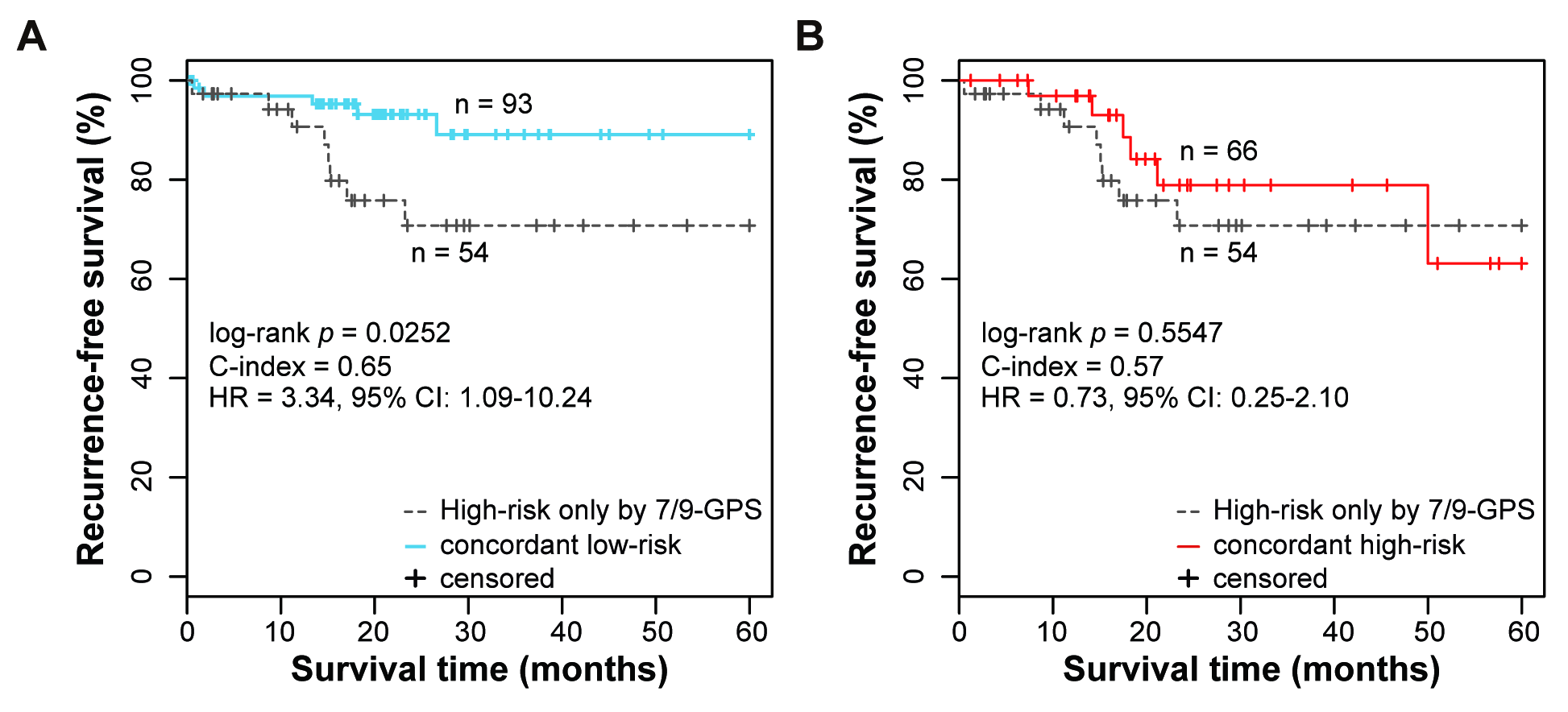


**Figure S2—The survival analyses of the high-risk samples identified by 7/9-GPS but not by 9-GPS and the risk samples concordantly by 7/9-GPS and 9-GPS in TCGA. (A)** The Kaplan–Meier curves of RFS for the high-risk stage I samples identified by 7/9-GPS but not by 9-GPS and the low-risk stage I samples identified concordantly by 7/9-GPS and 9-GPS. **(B)** The Kaplan–Meier curves of RFS for the high-risk stage I samples identified by 7/9-GPS but not by 9-GPS and the high-risk stage I samples identified concordantly by 7/9-GPS and 9-GPS.


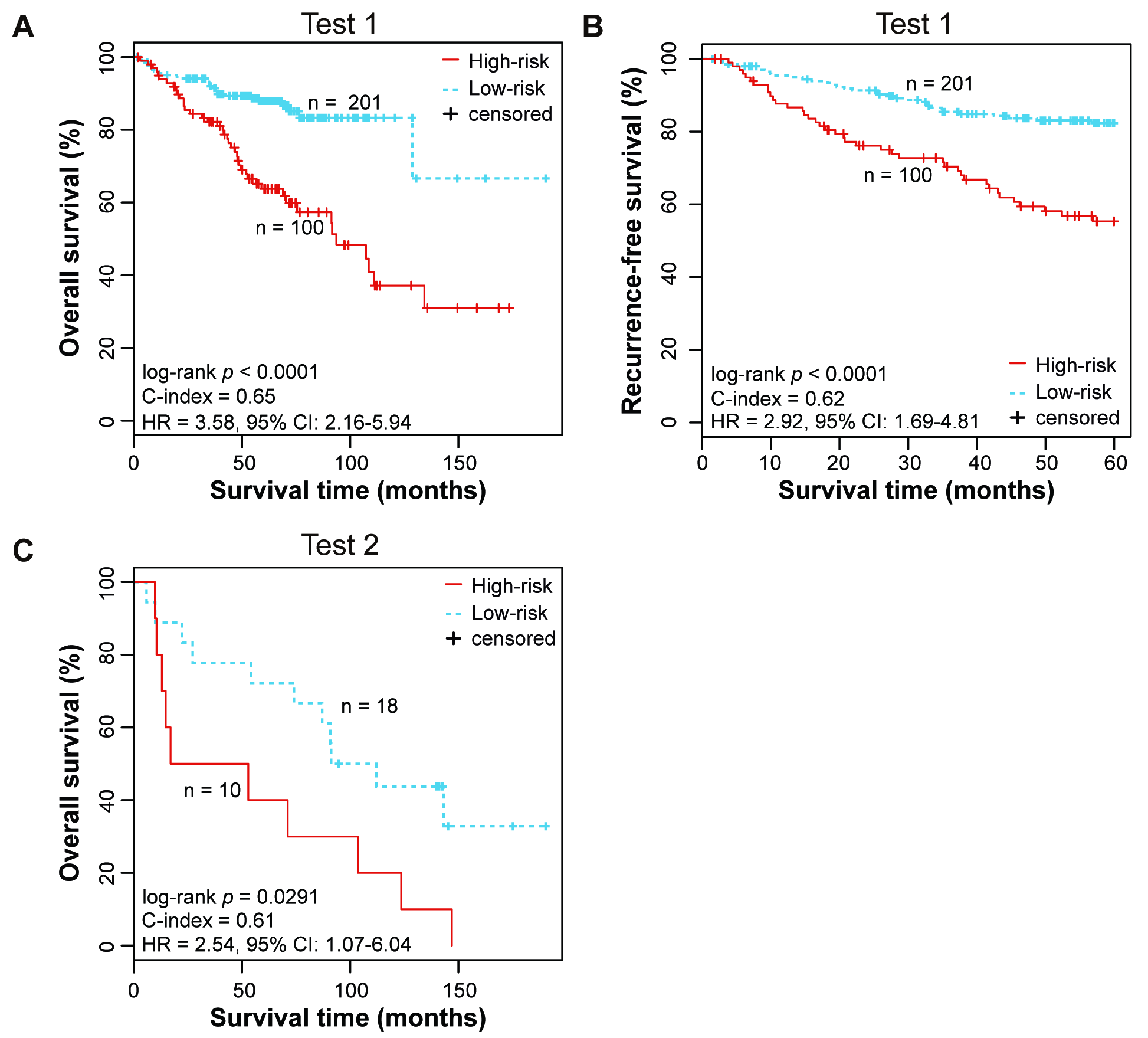


**Figure S3—Prognostic performance of 9-GPS based on the majority voting rule in two test data sets. (A)** The Kaplan–Meier curves of overal survival (OS) for 301 stage I samples in test 1. **(B)** The Kaplan–Meier curves of RFS for 301 stage I LUAD samples in test 1. **(C)** The Kaplan–Meier curves of OS for 28 stage I samples in test 2.


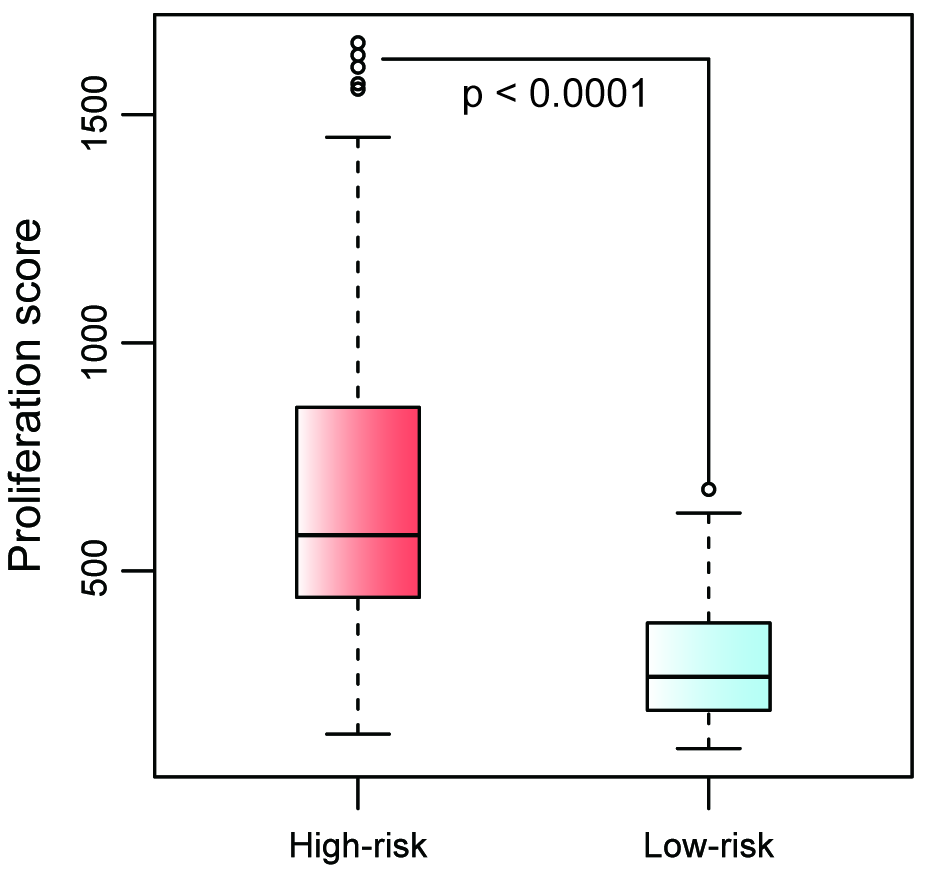


**Figure S4—The boxplot of proliferation scores in the high-risk and low-risk samples identified by 7/9-GPS, respectively.**


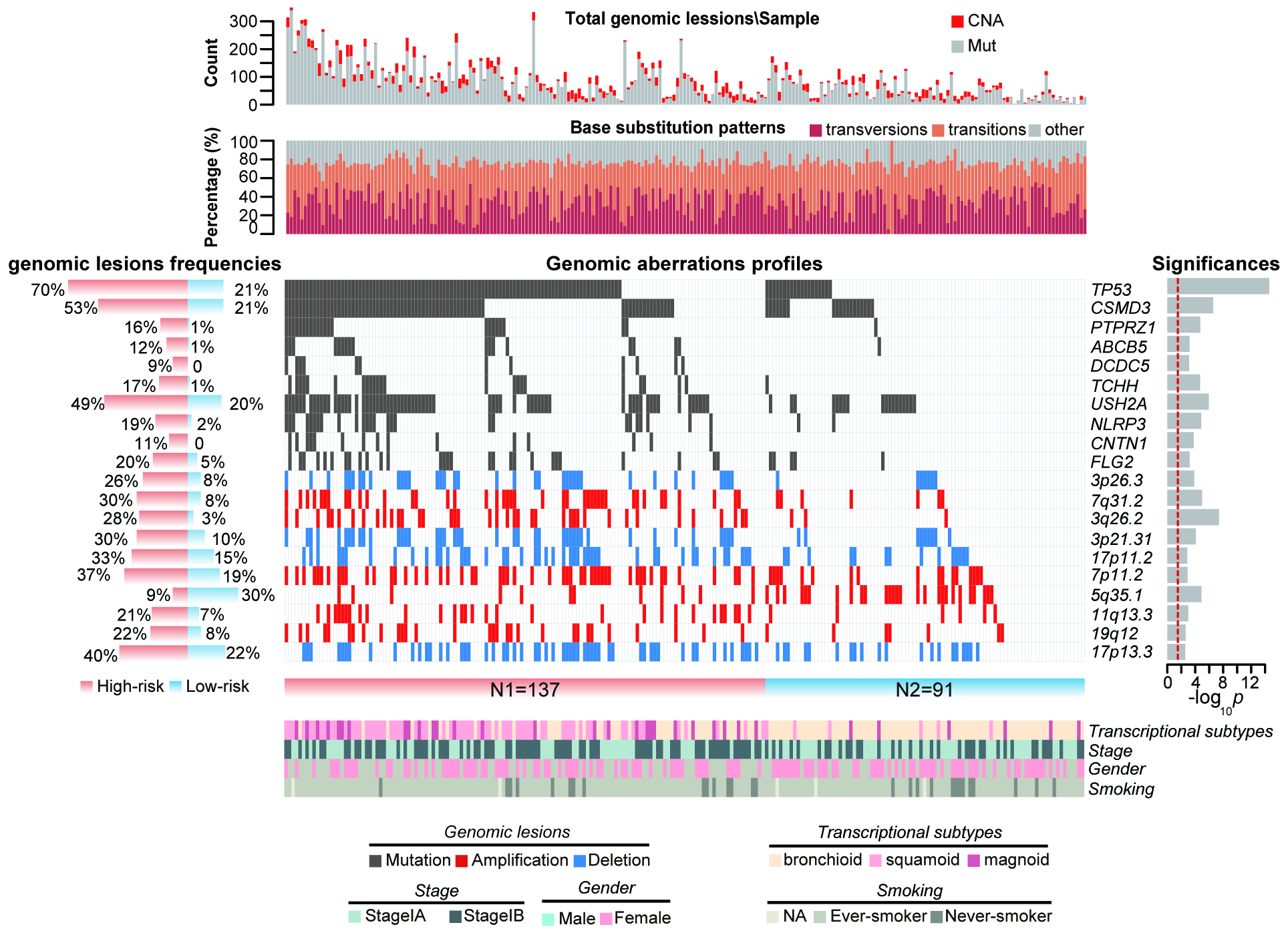


**Figure S5—****The genomic** **characteristics between the high- and low-risk groups predicted by 7/9-GPS in stage I LUAD patients.** The 45 of the 48 genomic lesions (Supplementary Table 4) characterizing the difference between the reclassified metastatic samples and non-metastatic samples also had significantly different mutation or CNA frequencies between the stage I high-risk and low-risk samples identified by 7/9-GPS (Fisher’s exact test, FDR<0.05). Some genomic lesions between the two risk groups including ten gene mutations and ten chromosome regions are displayed. All the 48 genomic lesions are displayed in Supplementary Table 5. The frequencies of the two risk groups with lesions are shown at the left and the significances of the frequency differences between the two groups are listed at the right. The clinical information for clinically diagnosed metastasis states (yes/no), stage, gender and smoking, and the total genomic lessions count, including mutation count and CNA count, mutation base substitution patterns, transcriptional subtypes for 228 stage I samples are also demonstrated.


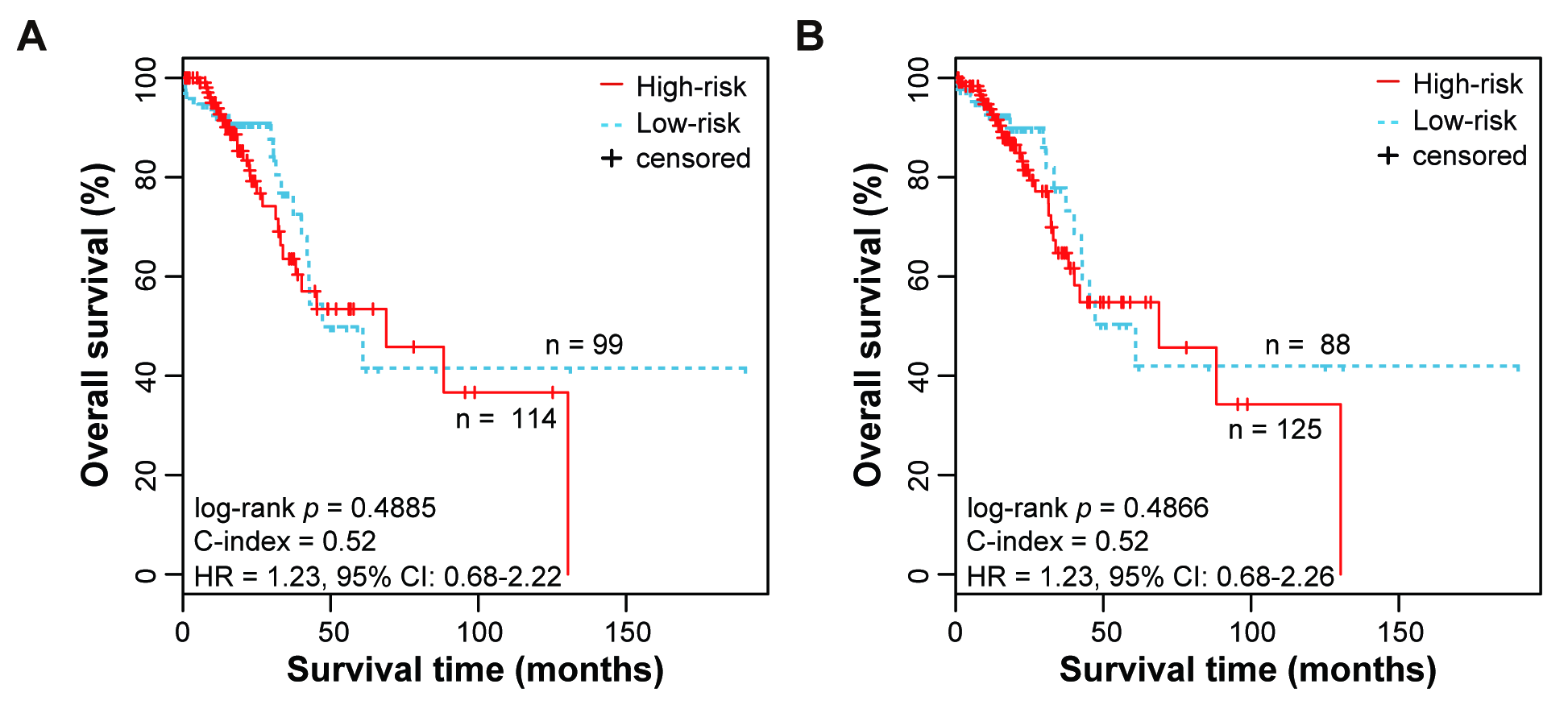


**Figure S6—Prognostic performance of quantitative gene expression signatures in 213 stage I lung adenocarcinoma samples in TCGA. (A)** Prognostic performance of malignancy-risk gene signature for OS of 213 stage I LUAD samples in TCGA. **(B)** Prognostic performance of 16-gene signature for OS of 213 stage I LUAD samples in TCGA.
